# Catalytic Asymmetric Hydroalkoxylation of C–C Multiple Bonds: Focus Review

**DOI:** 10.1021/acs.chemrev.1c00620

**Published:** 2021-12-03

**Authors:** Jennifer L. Kennemur, Rajat Maji, Manuel J. Scharf, Benjamin List

**Affiliations:** Max-Planck-Institut für Kohlenforschung, Kaiser Wilhelm-Platz 1, 45470 Mülheim an der Ruhr, Germany

## Abstract

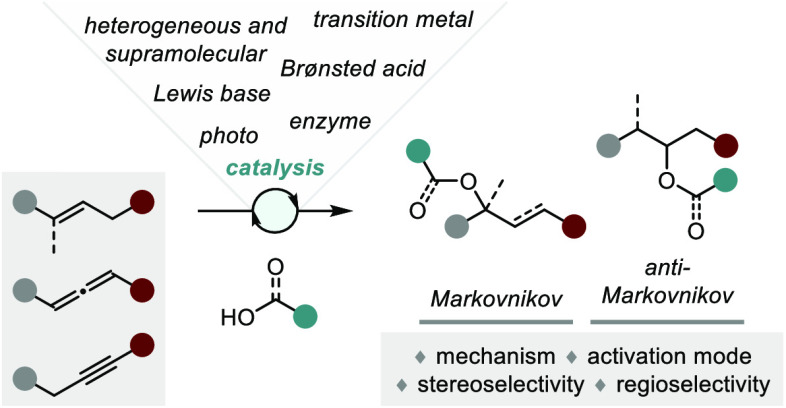

Asymmetric hydroalkoxylation of alkenes constitutes a redox-neutral and 100% atom-economical strategy toward enantioenriched oxygenated building blocks from readily available starting materials. Despite their great potential, catalytic enantioselective additions of alcohols across a C–C multiple bond are particularly underdeveloped, especially compared to other hydrofunctionalization methods such as hydroamination. However, driven by some recent innovations, e.g., asymmetric MHAT methods, asymmetric photocatalytic methods, and the development of extremely strong chiral Brønsted acids, there has been a gratifying surge of reports in this burgeoning field. The goal of this review is to survey the growing landscape of asymmetric hydroalkoxylation by highlighting exciting new advances, deconstructing mechanistic underpinnings, and drawing insight from related asymmetric hydroacyloxylation and hydration. A deep appreciation of the underlying principles informs an understanding of the various selectivity parameters and activation modes in the realm of asymmetric alkene hydrofunctionalization while simultaneously evoking the outstanding challenges to the field moving forward. Overall, we aim to lay a foundation for cross-fertilization among various catalytic fields and spur further innovation in asymmetric hydroalkoxylations of C–C multiple bonds.

## 1 Introduction

**Figure 1:**
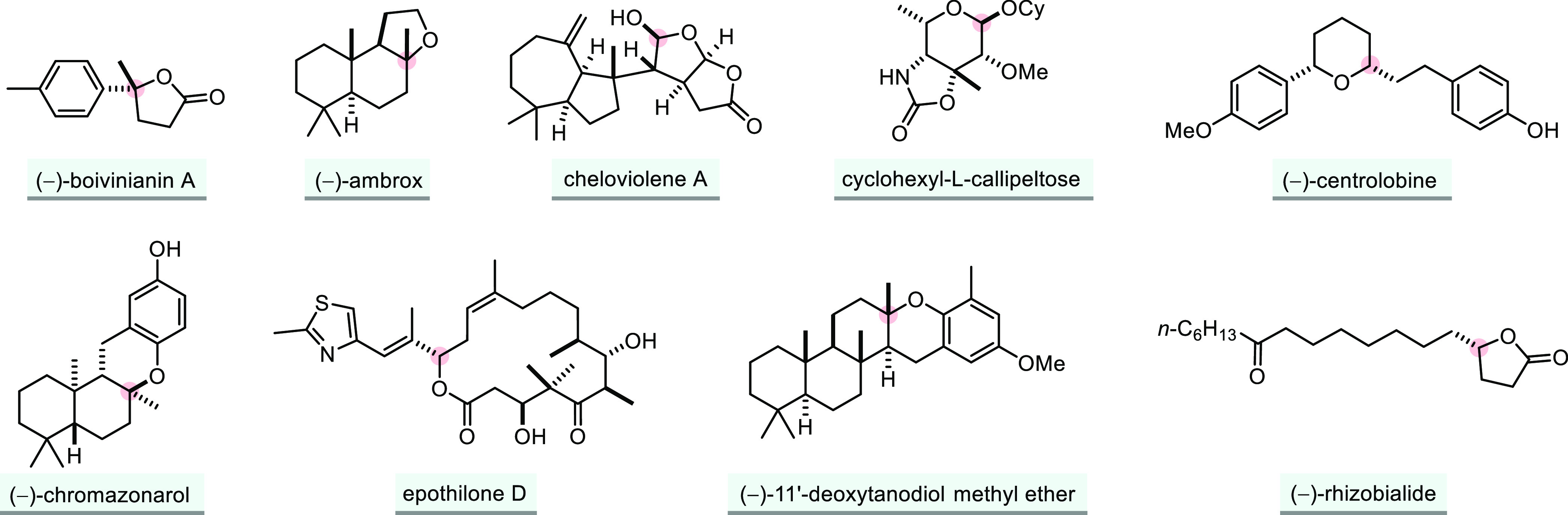
A survey of natural products or related structures prepared via stereoselective hydroalkoxylation or hydroacyloxylation.

Hydrofunctionalization of a C–C multiple bond provides an atom- and step-economical strategy to introduce structural and stereochemical complexity toward value-added chemicals and medicinally germane compounds.^1−5^ Given its widespread synthetic utility, translating this approach into asymmetric hydrofunctionalization methods has garnered significant attention from the catalysis community.^6^ In particular, stereoselective additions of alcohols, carboxylic acids, and water across C–C multiple bonds (i.e., hydroalkoxylation, hydroacyloxylation, and hydration) are attractive redox-neutral tools for generating stereoenriched ethers, acetals, esters (including lactones), and alcohols.^7−9^ Given the prevalence of such motifs in bioactive compounds,^10^ stereoselective additions of O–H bonds across C–C multiple bonds have enabled access to key chemical synthons en route to natural products or derivatives thereof (Figure 1).^11−18^ However, catalyst-controlled enantioselective methods have remained relatively scarce compared to other asymmetric hydrofunctionalizations (e.g., hydroamination).^19^ We attribute the dearth of asymmetric methods to intrinsic challenges associated with such transformations rather than a lack of compelling interest to efficiently access the corresponding synthetically important functionalities. For example, akin to hydroamination reactions,^20^ while additions of O–H bonds to C–C double bonds tend to be thermodynamically feasible or thermoneutral (e.g., Δ*G*° = −4.1 kcal/mol for the hydration of 2-butene in H_2_O^21^), they are typically impeded by relatively high kinetic barriers. Further, garnering reactivity with relatively weak oxygen nucleophiles under reaction conditions conducive to asymmetric induction is challenging (*N* (MeNH_2_) = 15.19 in MeCN;^22^*N* (MeOH) = 6.86 in MeCN^23^), particularly with weakly Lewis basic alkenes.

**Scheme 1:**
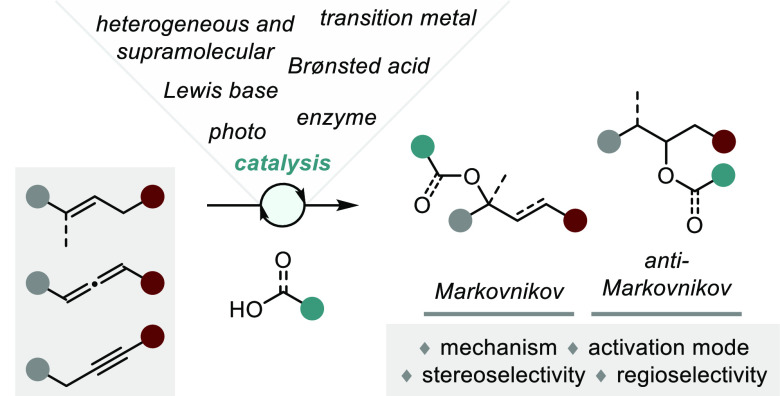
General Overview of the Topics Discussed in This Review

Gratifyingly, as a result of key developments in a broad range of catalytic fields (transition metal catalysis, photocatalysis, organocatalysis, enzyme catalysis, heterogeneous catalysis, Lewis base catalysis), enantioselective additions of O–H bonds to unsaturated molecules have evolved considerably in recent years (Scheme 1). Building upon these successes, we find it timely to examine the current state-of-the-art research in catalyst-controlled stereoselective hydroalkoxylation while also considering potential growth areas for the future. In this review, we specifically aim to provide readers with a broad and encompassing overview of the diverse assortment of catalytic strategies employed in this developing field. Given the highly relevant mechanistic overlap, we also include several examples of asymmetric hydroacyloxylations and hydrations as well as cycloisomerizations involving the addition of an O–H group across a polyene scaffold. While we cover transformations of a broad range of C–C multiple bonds (alkenes, alkynes, allenes, and enol ethers), additions to alkenes bearing an electron-withdrawing group (Michael additions) are beyond the scope of this review. We encourage interested readers to consult more focused reviews on that topic.^24,25^

Central to the organization of the review, we categorize the reported literature based on the underlying reaction mechanism/catalytic activation mode. As such, we have broadly classified these reactions in seven different categories (sections 2–8). In each subfield, we provide a general overview of the methodology, focusing on the general principles governing regio- and/or stereoselectivity as well as specific challenges encountered for a given approach. Further, we have placed a special emphasis on the accompanied physical-organic and theoretical analysis that has played an important role in providing molecular level details into the mechanism and identify the existing bottlenecks that warrant further developments. Despite our earnest effort to categorize these reactions based on the underlying mechanism, overlap between subfields and/or a lack of conclusive experimental evidence can convolute unambiguous assignments. When relevant, we note such gray areas throughout our discussions. We conclude this review by highlighting some outstanding challenges and identifying potential areas of improvement that could provide an inspiration for future studies.

## 2 Transition Metal Catalysis

Owing to the versatile reactivity of metal π complexes, transition-metal catalysis has provided innumerable platforms for hydrofunctionalization reactions of C–C multiple bonds, including hydroamination, hydroformylation, and hydroboration reactions, among others.^1−5,26^ In recent years, considerable attention has been devoted to the employment of chiral ligand scaffolds and/or chiral anions to effect asymmetric variants of such processes. Interestingly, however, relative to other hydrofunctionalization strategies, transition metal-catalyzed asymmetric additions of oxygen nucleophiles to C–C multiple bonds are starkly underdeveloped.

**Scheme 2:**
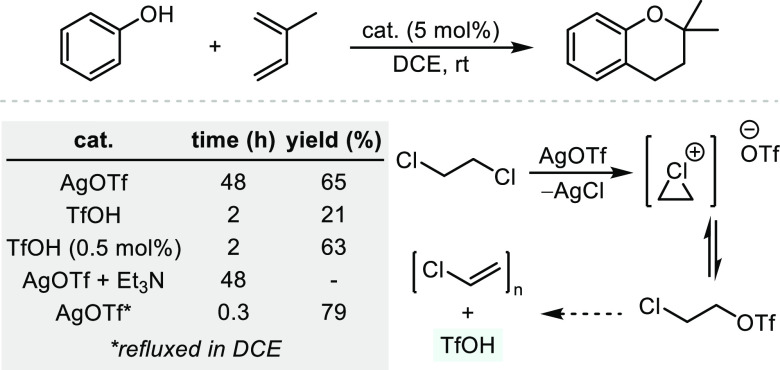
Hidden Brønsted Acid Catalysis

It has been suggested that asymmetric hydroalkoxylations are particularly challenging for transition-metal systems due to *hidden Brønsted acid catalysis*, i.e., the propensity of metal complexes to release competent Brønsted acids that are responsible for nonasymmetric activity.^27^ In fact, it has been demonstrated that common conditions used for metal-catalyzed hydroalkoxylation lead to the formation of trifluoromethanesulfonic acid (triflic acid, TfOH), a strong Brønsted acid (p*K*_a_ = −14.3 ± 2.0 in DMSO^28^) known to effect both intra- and intermolecular hydroalkoxylations.^29,30^ For example, the Hintermann group has shown that heating AgOTf in chlorinated solvents irreversibly forms TfOH (detected by ^19^F NMR spectroscopy), which the authors demonstrate as the active catalytic species in a phenol–isoprene cyclization (Scheme 2).^27^ The authors provide additional strong evidence that an early example of an asymmetric hydroalkoxylation using Cp*RuCl_2_ in the presence of AgOTf and a chiral bisphosphine ligand in toluene^31^ is catalyzed by in situ formed TfOH and disclose that the reported enantioselectivities are not reproducible (not depicted).

As such, it is imperative that hidden acid catalysis be considered during the development of asymmetric hydroalkoxylations and complexes bearing more basic ligands/anions might be preferred over triflates. As Hintermann points out, control reactions that simply replace metal triflates with equal loadings of TfOH and draw conclusions based on quantitative or qualitative differences in reactivity profiles can lead to a false impression that Brønsted acids are not involved in the main pathway of catalysis.^27^ Oftentimes, hidden acids can have advantages over pure TfOH and therefore such experiments can be misleading. For example, in a hydroalkoxylation reaction of dicyclopentadiene using Cu(OTf)_2_, the [Cu] species suppresses polymerization of the nucleophilic partner (2-hydroxyethyl methacrylate), and therefore the control experiment with pure TfOH resulted in gelation of the reaction mixture and poor overall yields of the desired product.^32^ Nevertheless, the authors provide strong evidence that TfOH is indeed the catalytically active species, underscoring the importance of well-designed control experiments.

To this end, protocols developed by Hintermann that deliberately generate hidden acids are encouraged as benchmark control experiments in reactions involving metal triflates.^27^ Additionally, one of the strongest arguments that a transition-metal complex is responsible for catalytic reactivity is the induction of high levels of enantioselectivity when employing a chiral ligand scaffold. We will herein delineate transition-metal-catalyzed methods that achieve enantioselectivity in hydroalkoxylation/acyloxylation reactions. When relevant, we will discuss possible hidden acid catalysis and highlight relevant mechanistic probes.

**Scheme 3:**
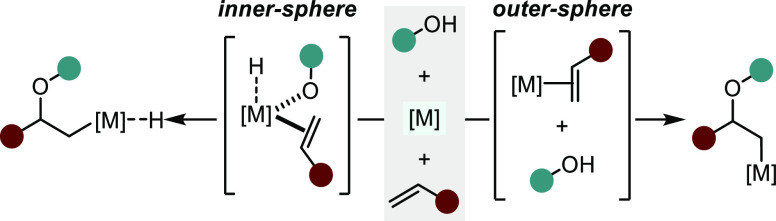
Two Mechanistic Subclasses of Transition Metal-catalyzed Hydroalkoxylation

This section has been divided into two mechanistically distinct subclasses: (1) metal complexes that proceed through an inner-sphere mechanism (Scheme 3, left) and (2) those proceeding via an outer-sphere nucleophilic attack by the oxygenated species (Scheme 3, right).^7^ Whether a process follows an inner- or outer-sphere pathway can have important implications on the stereochemical outcome of a reaction and is dependent on a number of factors, including the nature of the unsaturated substrate, the transition metal involved and its corresponding coordination geometry, the ligand scaffold, the nucleophilic species, and the general reaction conditions. Recent studies have revealed relatively low barriers of migratory insertion of alkenes into M–OR bonds and have suggested that some methods previously considered outer-sphere might in fact proceed through inner-sphere mechanisms.^33^ This distinction can be especially ambiguous in Pd-catalyzed transformations. Hence, we recognize the ever-evolving nature of mechanistic postulation and have organized these methods based on current proposals.

### 2.1 Inner-Sphere Mechanism

#### 2.1.1 Alkenes

**Scheme 4:**
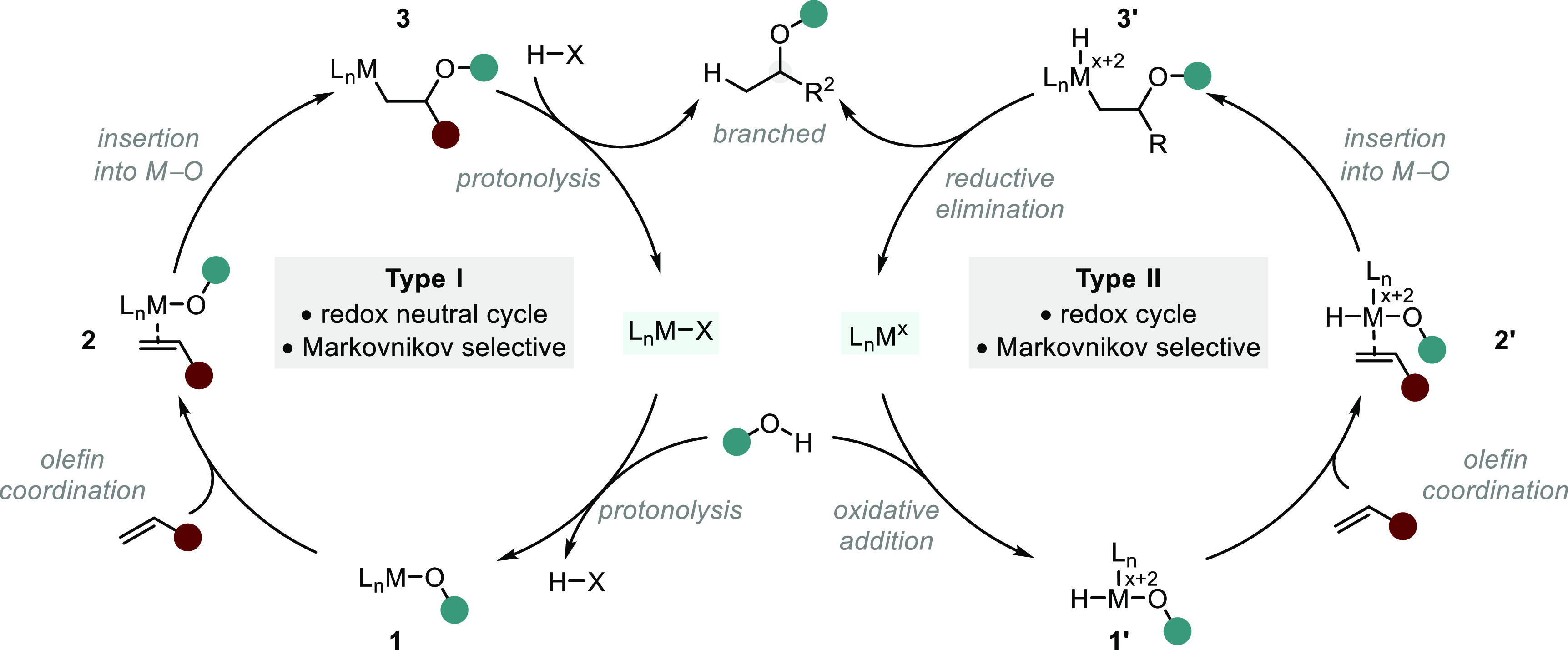
Two General Processes Describing Inner-sphere Mechanisms of Hydroalkoxylation

Metal-catalyzed additions of O–H bonds across C–C π bonds commonly proceed through an inner-sphere mechanism in the presence of electron-rich metals (e.g., Rh, Ir).^7^ Herein, we will consider two general mechanistic scenarios that govern hydroalkoxylations/acyloxylations of alkenes proceeding through nucleophilic activation (Scheme 4).^34^ Type I describes a redox neutral cycle initiated by protonolysis of a M–X bond to yield an alkoxy/acyloxy metal complex (M–OR, **1**). Alkene coordination and subsequent 1,2-insertion into the M–OR bond results in the branched product following protonolysis. Alternatively, type II mechanisms proceed via oxidative addition into the O–H bond to form an alkoxy/acyloxy-hydrido metal complex (H–M–OR, **1′**). Alkene coordination and subsequent 1,2-migratory insertion into the M–OR bond provides the terminal alkyl-hydrido metal complex (**3′**), which undergoes reductive elimination to form the Markovnikov adduct and regenerate the active catalyst. In either case, the regioselectivity of migratory insertion is typically dictated by the formation of the kinetically favored metal–alkyl complex (**3** or **3′**), in which the metal resides at the less encumbered carbon. Alternatively, from complex **2′**, migratory insertion into the M–H bond could also be envisaged; however, insertion of alkenes into the M–H bonds of such complexes are rare and, to the best of our knowledge, are so far limited to examples proceeding through kinetically favored metallocycles (vide infra).^8^ As a result, transition-metal mediated hydroalkoxylation/acyloxylation of alkenes generally provides the branched adduct in high regioselectivities and pathways to linear adducts prove challenging and largely undiscovered. Notably, M–H insertions have been demonstrated in an anti-Markovnikov hydroalkoxylation of porphyrin-based Rh-alkyl substrates; however, these reactions are stoichiometric in Rh.^35^

**Scheme 5:**
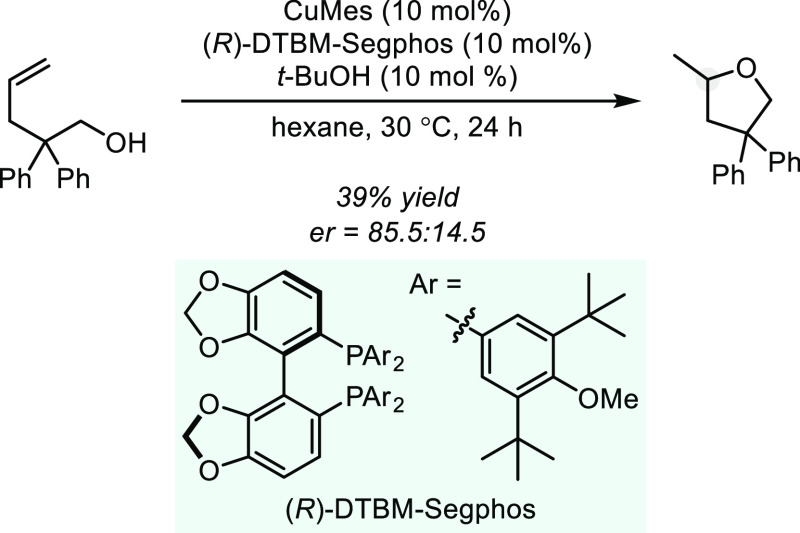
Cu-Catalyzed Hydroalkoxylation of 2,2-Diphenylpent-4-en-1-ol

In 2015, Ohmiya and Sawamura et al. reported an (*R*)-DTBM-Segphos-based Cu(I)-catalyzed asymmetric cyclization of 2,2-diphenylpent-4-en-1-ol to form the corresponding tetrahydrofuran with an er up to 85.5:14.5 in relatively low yield (Scheme 5).^36^ The authors propose a catalytic cycle consistent with type I, in which mesityl-Cu(I) undergoes protonolysis to form an alkoxy Cu(I) complex (and mesitylene), which proceeds through subsequent migratory insertion and finally protonolysis of the corresponding alkyl–M bond to form the cyclized product. Asymmetric induction is demonstrated with a single substrate and reactivity is generally limited to β,β-disubstituted alkenols containing a terminal alkene.

**Scheme 6:**
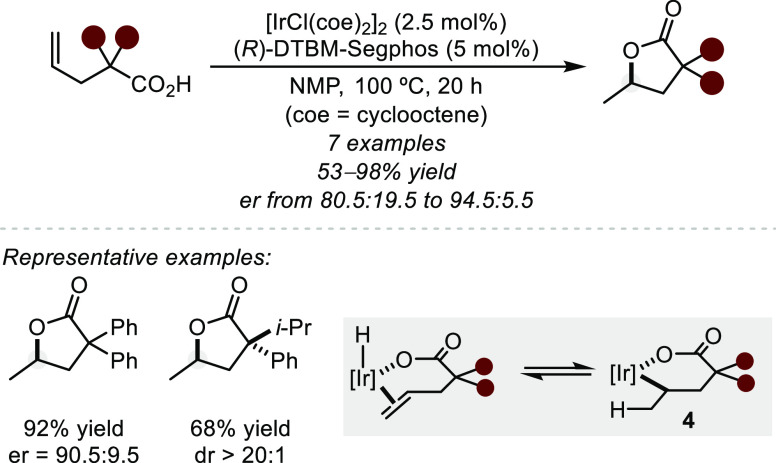
Ir-Catalyzed Hydroacyloxylation toward γ-Lactones

Similarly, the Nishumura group developed an Ir-catalyzed hydroacyloxylation of geminally disubstituted alkenoic acids using (*R*)-DTBM-Segphos to yield γ-lactones with good levels of enantioselectivity (Scheme 6).^37^ Stoichiometric studies of the precatalyst in the presence of substrate revealed the formation of a hydrido iridium (Ir–H) complex, providing evidence that the reaction proceeds via an oxidative addition pathway. However, in contrast to the depicted type II mechanism, the authors propose a 2,1-insertion into the M–H bond to form the kinetically favored 6-membered iridacycle (**4**), ultimately yielding the Markovnikov adduct following reductive elimination. Notably, in each of the above examples, chiral ligands derived from 3,5-di-*tert*-butyl-4-methoxyphenyl (DTBM)-substituted bisphosphines prove dramatically superior at enantioinduction compared to other examined ligand scaffolds.

**Scheme 7:**
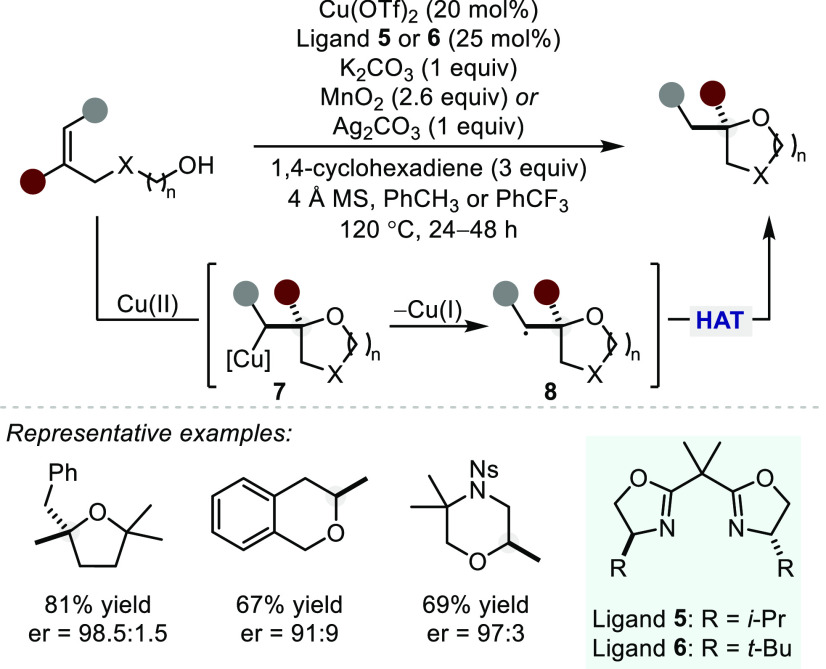
Cu(BOX)-Catalyzed Hydroalkoxylation

More recently, Chemler and co-workers described an asymmetric cyclization of a range of 4- and 5-substituted alkenols catalyzed by a chiral Cu(II)-bis(oxazoline) (BOX) complex to yield the corresponding 5- and 6-membered cyclic ethers with moderate to high levels of enantioselectivity (Scheme 7).^38^ The authors suggest that the catalytic cycle commences with anion exchange of the Cu–OTf precatalyst with the alkenol followed by ligand dissociation to form a cationic alkoxy Cu intermediate. An enantiodetermining oxycupration subsequently provides an alkyl Cu species (**7**) that undergoes homolytic C–Cu bond cleavage to render an alkyl radical (**8**). A subsequent hydrogen atom transfer (HAT) from 1,4-cyclohexadiene yields the product and a stoichiometric oxidant (MnO_2_ or Ag_2_CO_3_) regenerates the active Cu(II) species. The addition of K_2_CO_3_ seemingly prevents the formation of TfOH, suppressing hidden acid catalysis and enabling high levels of enantioinduction. However, the enantioselectivity is essentially unaffected without added K_2_CO_3_ (using either MnO_2_ or air as the oxidant), albeit with reduced yields. This experiment suggests that, if TfOH does form, the Cu complex outcompetes the acid pathway in the formation of the product (though the authors do not comment on side reactivity).

While each of the above methods have provided access to a number of enantioenriched cyclic ethers and lactones, there is a clear pattern of substrate engineering tailored to the Thorpe–Ingold effect, such that the reactivity scope of transition-metal catalyzed intramolecular hydroalkoxylations remains inherently limited. Efforts toward the development of asymmetric methods employing simpler alkenols/alkenoic acids are encouraged. For example, Marks and co-workers have reported organolanthanide-catalyzed intramolecular Markovnikov hydroalkoxylations of a diverse range of alkenols, including the cyclization of simple pent-4-en-1-ol to 2-methyltetrahydrofuran.^39^ While a free TfOH-catalyzed process as a major pathway has tentatively been ruled out, asymmetric variants of these methods have not been reported.

**Scheme 8:**
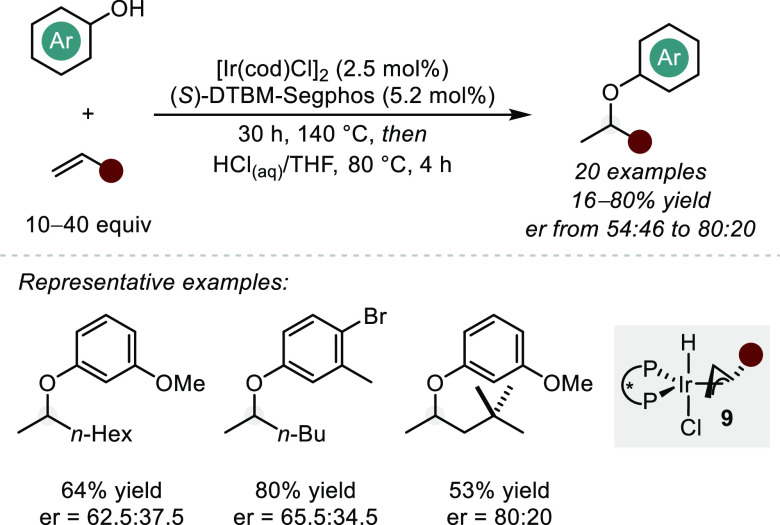
Ir-Catalyzed Intermolecular Hydroalkoxylation of Simple Alkenes

Another significant challenge in the development of transition metal-catalyzed asymmetric hydroalkoxylations and hydroacyloxylations is their extension to intermolecular systems. The relatively low basicity of simple C–C double bonds renders the formation of metal π complexes with alkenes extremely challenging and reactivity typically depends on either high alkene loadings or Lewis basic groups tethered to the alkenic unit.^40,41^ In 2013, the Hartwig group impressively showed that the combination of Ir and (*S*)-DTBM-Segphos catalyzes the addition of phenols to structurally simple alkenes (solvent quantities) in good yields and poor to moderate enantioselectivities (Scheme 8).^19^ A number of experiments (including the use of chiral ligands and measurable enantioinduction with all substrates) provide strong evidence that the reaction is indeed catalyzed by the metal complex and that hidden Brønsted acid catalysis is either limited or completely suppressed. The kinetic profile of the reaction is consistent with a type II redox mechanism and a turnover-limiting oxymetalation step via an alkoxy-hydrido Ir(III) complex. As with the aforementioned intramolecular examples, this method highlights the privileged nature of the DTBM-derived ligand scaffolds, as all other evaluated ligand classes provided little to no reactivity.

This method reveals two additional challenges facing transition-metal-catalyzed hydroalkoxylations. First, alkene isomerization leads to unreactive internal C–C double bonds, further necessitating high loadings of the starting terminal alkene. Notably, alkene isomerization is more significant in this hydroalkoxylation with phenol than in a similar Ir-catalyzed hydroamination published by the same authors.^42^ In this hydroalkoxylation case, the phenol unit is not basic enough to stabilize the Ir complex and, consequently, an 18 electron Ir(III) allyl hydride complex is the catalyst resting state (Scheme 8, structure **9**), which leads to increased isomerization. A second challenge is that the alkyl Ir complex following oxymetalation is prone to β-hydride elimination and the corresponding enol ethers are observed in moderate quantities. Further, because the alkene is the terminal reductant for the enol ether side product, the corresponding saturated alkanes are also observed. This represents a general challenge in similar hydroamination strategies, as analogous enamines are typically observed.^42,43^ Despite these drawbacks, this method demonstrates the robust capacity of transition-metal systems to engage weakly basic and unfunctionalized alkenes and effect asymmetric hydroalkoxylation reactions. We anticipate improved chemo- and enantioselectivities in future developments.

#### 2.1.2 Allenes and Alkynes

The increased Lewis basic nature of allenes and alkynes compared to alkenes increases the propensity of these functionalities to form metal π complexes and subsequently participate in organometallic reactions. Additionally, the degree of unsaturation that remains following metal-catalyzed reactions of allenes and alkynes has been shown to stabilize metal complexes through π-coordination and accelerate catalytic processes.^44^ We will herein describe transition-metal-catalyzed asymmetric hydroalkoxylations and hydroacyloxylations of allenes and alkynes involving inner-sphere attack of the nucleophile, a field so far dominated by Rh catalysis. For the methods described in this section, alkynes and allenes proceed through a common catalytic intermediate, and therefore we will discuss these functionalities in parallel.

**Scheme 9:**
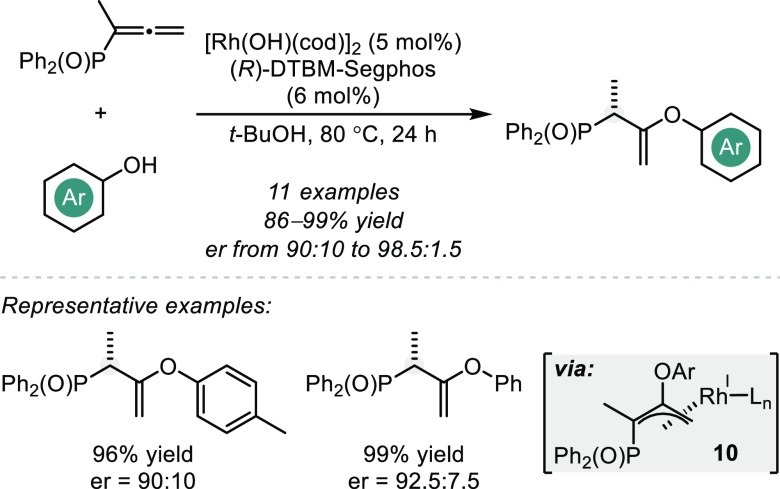
Intermolecular Rh-catalyzed Hydroalkoxylation of Diphenylphosphinylallenes

The seminal report on intermolecular asymmetric hydroalkoxylations of allenes was published by Nishimura and Hayashi et al. in 2009 and describes a DTBM-Segphos-based Rh-catalyzed addition of phenols to diphenylphosphinylallenes to yield vinyl ethers in high yields and enantioselectivities (Scheme 9).^45^ On the basis of ^1^H and ^31^P NMR studies, the authors propose that the reaction proceeds via protonolysis of the Rh precatalyst to form a phenoxorhodium species that reacts with the allene substrate to concomitantly forge a C–O bond and generate a π-allylrhodium intermediate (**10**). Protonation of the π-allyl system with phenol regenerates the active catalyst and yields the enantioenriched enol ether product. While this method provides precedence for an enantioselective intermolecular Rh-catalyzed hydroalkoxylation of allenes, the dependency on diphenylphosphoryl substituents imposes limitations on its synthetic applicability and elicits the development of more general catalytic solutions.

To this end, Trost and Yamamoto published a series of methods throughout the 1990s on Pd-catalyzed additions of nucleophiles (including carboxylic acids and alcohols) to alkyl-substituted allenes and alkynes.^46−53^ A key mechanistic feature of these reactions is the formation of a Pd π-allyl intermediate, which undergoes a regioselective outer-sphere nucleophilic attack to yield linear allylic products. A few enantioselective Pd-catalyzed hydroalkoxylations and hydroacyloxylations have been developed based on this work and will be discussed in section 2.2.1. Alternatively, Evans and others have shown that Rh-catalyzed allylic substitution reactions (which proceed through analogous Rh π-allyl intermediates) result in high levels of regioselectivity toward *branched* allylic compounds.^54,55^ As such, Breit recognized that Rh-catalyzed nucleophilic additions to alkynes and allenes could offer a platform for asymmetric syntheses of branched allylic compounds, avoiding stoichiometric byproducts and offering complementary regioselectivity to related Pd-catalyzed transformations.^56^

**Scheme 10:**
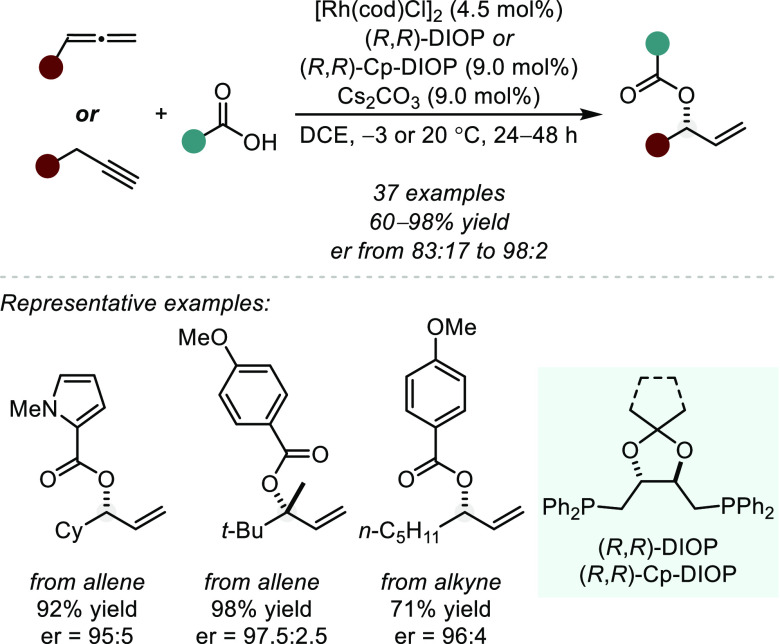
Rh-Catalyzed Hydroacyloxylations of Allenes and Alkynes

Accordingly, in 2011, Breit and co-workers described a highly enantioselective Rh-catalyzed hydroacyloxylation of terminal allenes to form allylic esters using (*R*,*R*)-DIOP as a chiral ligand (Scheme 10, *from allene*).^57^ Notably, the method is tolerant of free alcohols and could be used to form a quaternary stereocenter with excellent enantioselectivity. Additionally, the Breit group has shown the robustness of this method with the formation of various macrocyclic scaffolds, as well as key intermediates en route to several natural products.^58−63^

**Scheme 11:**
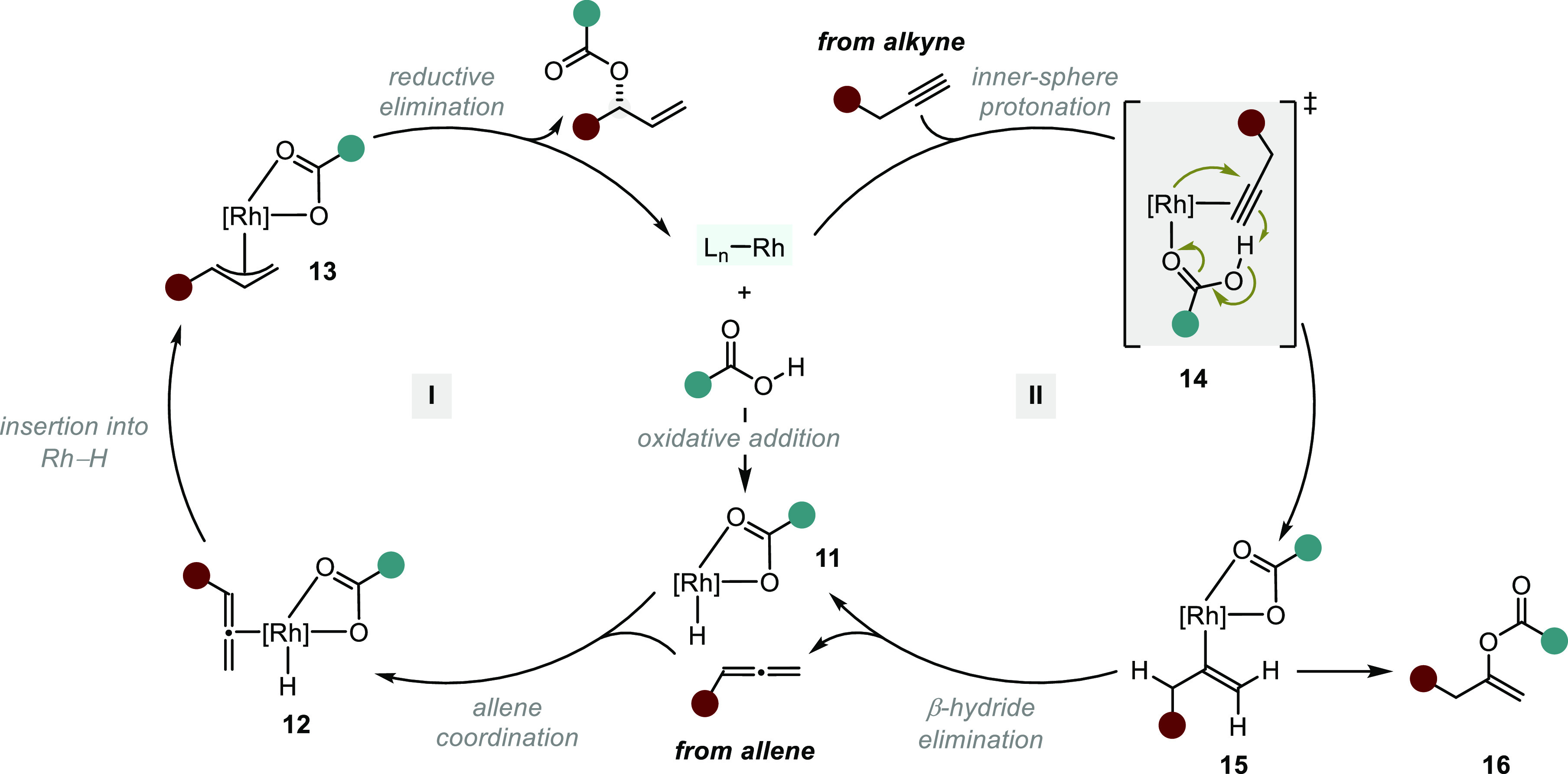
Mechanistic Proposal for Rh-Catalyzed Hydroacyloxylations of Allenes and Alkynes

The authors propose that the reaction proceeds via an oxidative addition into the carboxylic acid to generate acyloxy-hydrido rhodium complex **11**, which, following allene coordination, undergoes a hydrometalation to generate Rh π-allyl intermediate **13**. An ensuing reductive elimination yields the branched allylic ester and regenerates the Rh(I) catalyst (Scheme 11, cycle I). However, the authors have not ruled out an outer-sphere nucleophilic attack.

Breit and co-workers later expanded this methodology by capitalizing on the well-established proclivity of alkynes to isomerize to allenes in the presence of Rh–H complexes.^64^ On the basis of their previous work using an achiral phosphine ligand,^65^ Breit et al. developed a Rh(I)/(*R*,*R*)-Cp-DIOP-catalyzed enantioselective hydroacyloxylation of alkynes (Scheme 10, from alkyne).^66^ Mechanistic studies, including DFT calculations and extensive experimental investigations, suggest that the isomerization pathway proceeds via an inner-sphere protonation of the terminal alkyne by a Rh-coordinated carboxylic acid, leading to the formation of **15** (Scheme 11, cycle II).^67^ This is mechanistically distinct from related Pd-catalyzed isomerizations, which are thought to proceed through hydrometalation pathways. From complex **15**, β-hydride elimination releases an allene, and concurrently generates the acyloxy-hydrido complex (**11**) and enters the aforementioned catalytic cycle I. Alternatively, reductive elimination of complex **15** yields *gem*-enol ester **16**, a side product observed in relatively low quantities.

**Scheme 12:**
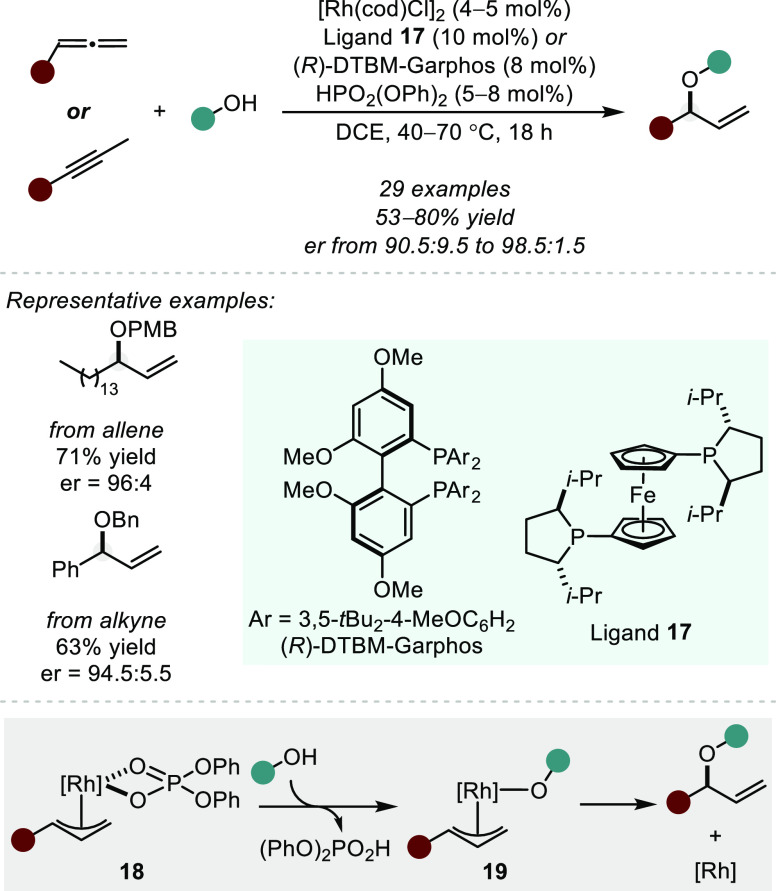
Rh-Catalyzed Hydroalkoxylation of Allenes and Alkynes

In 2016, the same group demonstrated a Rh(I)/diphenyl phosphoric acid-catalyzed enantioselective hydroalkoxylation of allenes and internal Me-substituted alkynes (Scheme 12).^68^ A wide range of simple and functionalized alcohols were tolerated, including a later addition of *N*-hydroxyphthalimides,^69^ which undergo facile cleavage to furnish enantioenriched allylic alcohols. The mechanism is similar to that proposed for the addition of carboxylic acids; however, in this case, the phosphoric acid is used to generate the Rh–H intermediate and subsequently form the electrophilic Rh π-allyl species **18**. Anion exchange yields the alkoxy Rh π-allyl intermediate **19**, followed by reductive elimination to furnish the branched allylic ether product. The authors can again not rule out the possibility of an outer-sphere nucleophilic attack of the alcohol on the Rh π-allyl species. We wonder if enantioinduction would be observed using a chiral phosphoric acid in combination with an achiral phosphine ligand, which could probe the inner- vs outer-sphere nature of this step.

### 2.2 Outer-Sphere Mechanism

**Scheme 13:**
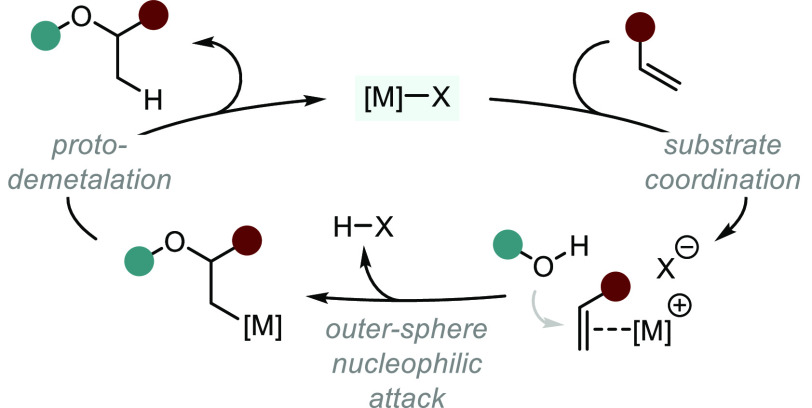
General Outer-sphere Mechanism Governing Lewis Acid-catalyzed Hydroalkoxylations

Most transition metals, by virtue of their low-lying vacant d-orbitals, are able to bind carbon–carbon multiple bonds as π-Lewis acids. In contrast to inner-sphere hydrofunctionalizations that proceed via migratory insertion of a coordinated π-bond into a metal-alkoxide, nucleophilic addition can occur from outside the ligand sphere of the metal; an elementary step referred to as *outer-sphere attack* (Scheme 13). Following addition, the newly formed alkyl-metal bond is cleaved through protonolysis to regenerate the active catalyst and yield the hydroalkoxylation product. While enantioselectivity can be induced by a chiral neutral metal-bound ligand, an intrinsic challenge for asymmetric induction originates from the distal nucleophilic attack, a factor that can be increasingly problematic depending on the coordination geometry of the metal complex (vide infra). Alternatively, asymmetric counteranion directed catalysis (ACDC) has emerged as a powerful tool for enantioselective hydrofunctionalizations. In this section, we will elaborate on both concepts through the aid of selected examples. Here, we will exclusively discuss examples where the newly formed C–O bond is part of a carbon stereocenter. Additionally, numerous examples of desymmetrizing hydroalkoxylations and hydroacyloxylations can be found in the literature that render the transformation asymmetric only by creation of a stereocenter distal to the reactive site.^70−73^ These transformations therefore lie outside the objective of this review.

#### 2.2.1 Chiral Metal–Ligand Scaffolds

In this section, we will highlight selected hydrofunctionalizations that proceed via outer-sphere nucleophilic attack, where stereoselectivity is induced by a chiral, neutral, metal-bound ligand. We will discuss the examples in order of the corresponding Lewis acidic metal and will start with a mechanistically distinct type of catalysis.

**Scheme 14:**
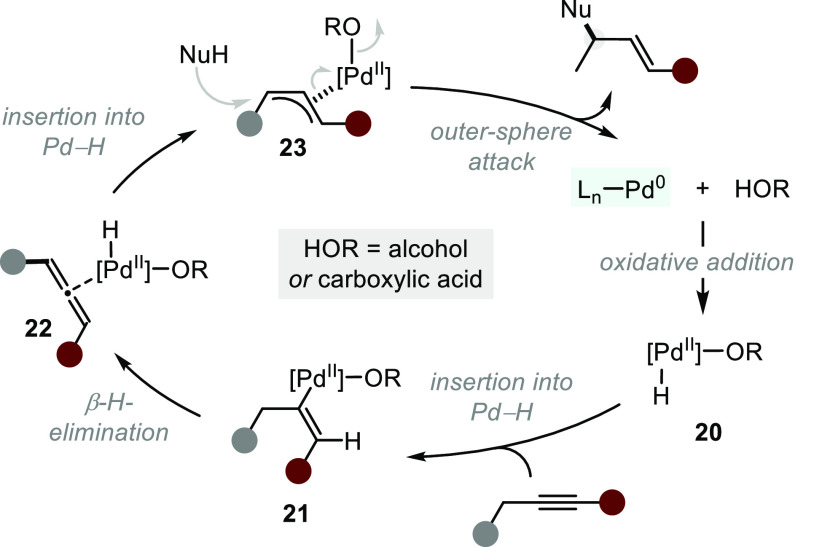
Mechanism of Palladium(0) Catalyzed Intramolecular Hydroalkoxylation of Alkynes

Similar to previously discussed inner-sphere Rh-catalyzed hydroalkoxylations and hydroacyloxylations of alkynes and allenes (section 2.1.2), outer-sphere hydrofunctionalization can be accomplished through Pd catalysis (Scheme 14).^46^ A key mechanistic feature is the formation of a palladium hydride **20** upon oxidative addition into an acidic O–H bond (alcohol or carboxylic acid). A series of hydropalladation and β-hydride elimination reactions lead to allyl-Pd complex **23** via an intermediate vinyl-palladium **21** and Pd–allene complex **22**. Ultimately, outer-sphere nucleophilic attack generates the product and, together with simultaneous or subsequent proton transfer, the initial Pd-hydride **20**. Asymmetric intramolecular alkoxide additions to allyl–palladium intermediates generated from allylic systems have also been reported in an overall oxidative fashion. We will not explicitly elaborate on these examples, as the overall transformation does not classify as a hydrofunctionalization.^74^

**Scheme 15:**
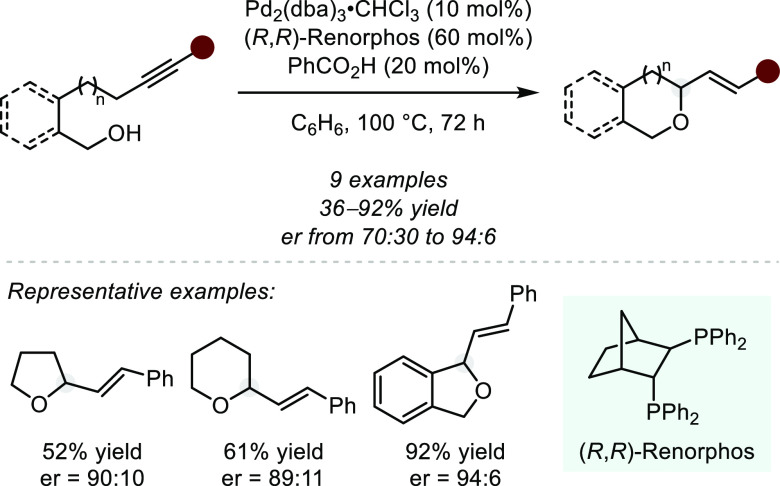
Palladium-catalyzed Intramolecular Hydroalkoxylation of Alkynes via Intermediate Allyl–Pd Complexes

On the basis of this concept, Yamamoto and co-workers achieved an intramolecular asymmetric hydroalkoxylation of alkynes (Scheme 15).^75^ Through the use of a chiral bisphosphine ligand, a palladium(0) source, and benzoic acid, an alkyne is converted to the corresponding allyl–palladium complex, which is ultimately intercepted by the alcohol. The authors were thus able to obtain furans, pyrans, as well as isochromanes in moderate to excellent yields and good enantioselectivity. Importantly, the origin of stereoinduction could not be unambiguously disclosed by the authors, as neither an allene, nor a 1,3-diene, both well-known precursors to Pd(II)-allyl complexes, could be reacted with high enantioselectivity. Computations suggest that the initial alkyne complexation already determines the enantioselectivity and that the corresponding Lewis acidic enantiopure palladium(II) complex remains closely associated to the π-cloud throughout the reaction mechanism.

**Scheme 16:**
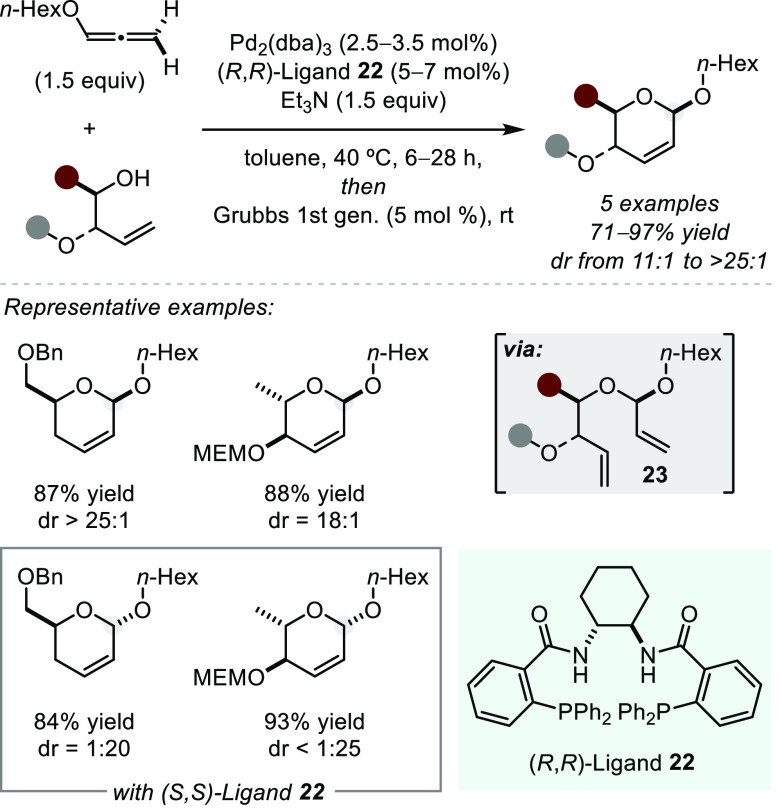
Palladium-catalyzed Asymmetric Intermolecular Hydroalkoxylation of Alkoxyallenes

An intermolecular palladium-catalyzed hydroalkoxylation via allyl–Pd intermediates was realized by Rhee et al. in 2014 (Scheme 16).^76^ Starting from terminal alkoxyallenes and simple enantiopure secondary alcohols, using Trost ligand **22**, the authors obtained acetals **23** that were in situ cyclized via olefin metathesis to the corresponding dihydropyrans. Under the influence of either enantiomer of the chiral ligand, opposite diastereomers of the products could be isolated with good selectivity, highlighting the catalyst control over diastereomeric reaction pathways. Notably, the products could be further elaborated to valuable glycoside building blocks by dihydroxylation. Additionally, Overman and co-workers later employed this method to access an enantiopure 3-chloro-5-alkoxybutenolide en route to several natural products.^16,77^ Further, Cao and co-workers later disclosed an asymmetric addition of phenols to alkoxyallenes using a Trost ligand in the presence of Pd(0) to yield acyclic *O*,*O*-acetals.^78^ On the basis of experimental evidence, the authors propose that the enantiodetermining step might be the insertion of the Pd–H bond into the allene system.

**Scheme 17:**
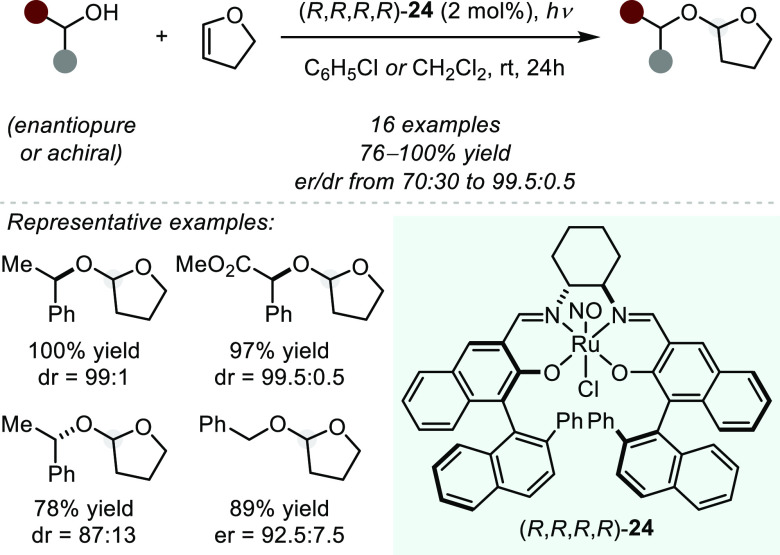
Ruthenium-catalyzed Asymmetric Intermolecular Acetal Formation

In a purely π-Lewis-acidic activation mode, an early and rare example of an intermolecular asymmetric hydroalkoxylation was reported by Katsuki and Nagano in 2002 (Scheme 17).^79^ The authors were able to achieve high stereoselectivity in the acetalization reaction of electronically biased primary or secondary alcohols with dihydrofuran under the influence of a chiral Ru(II)–salen complex. Notably, the reaction of either enantiomer of the starting material provided the THF-protected compounds in good diastereoselectivity, showcasing efficient catalyst-control even though a small matched–mismatched effect was observed. Mechanistically, catalyst **24** releases nitrous oxide upon irradiation,^80^ providing the active Lewis acidic ruthenium(III) catalyst with an open coordination site for substrate binding.

**Scheme 18:**
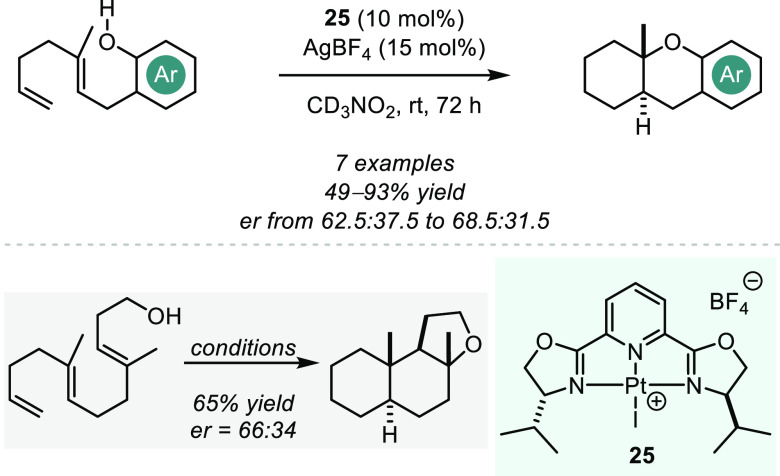
Platinum-catalyzed Asymmetric Polyene Cyclizations

Through the use of highly Lewis acidic platinum(II) pincer complexes, Gagné and Nguyen achieved a catalytic enantioselective cycloisomerization of polyenic compounds (Scheme 18).^81^ The Pt-bis(oxazoline)pyridine (PyBOX) precatalyst **25** is activated by silver tetrafluoroborate to generate a dicationic Pt(II) catalyst with noncoordinating anions enabling strong substrate binding and consequently high reactivity. In the reaction of several linear aromatic and aliphatic substrates, the authors were able to obtain cycloisomerized products in good to excellent yields, however only with poor enantioselectivities. The authors propose a catalytic cycle that begins with Lewis acidic activation of the least substituted C=C bond via coordination to the (PyBOX)Pt^2+^.

After the cyclization cascade, the former OH group transfers a proton which rapidly protodemetalates the in situ generated alkyl-platinum intermediate and thus releases the catalyst. The putative mechanism is supported by deuterium labeling studies and other control experiments. It is of importance to state that these types of polyene cyclizations do not formally represent a hydroalkoxylation, as an OH moiety is not added across a single multiple bond. However, the reaction can be perceived as an extended hydroalkoxylation across multiple double bonds, resulting in ring formations. We therefore include polyene cyclizations in this discussion for their mechanistic relevance and as a means to highlight catalytic approaches that could enable future progress in the field.

**Scheme 19:**
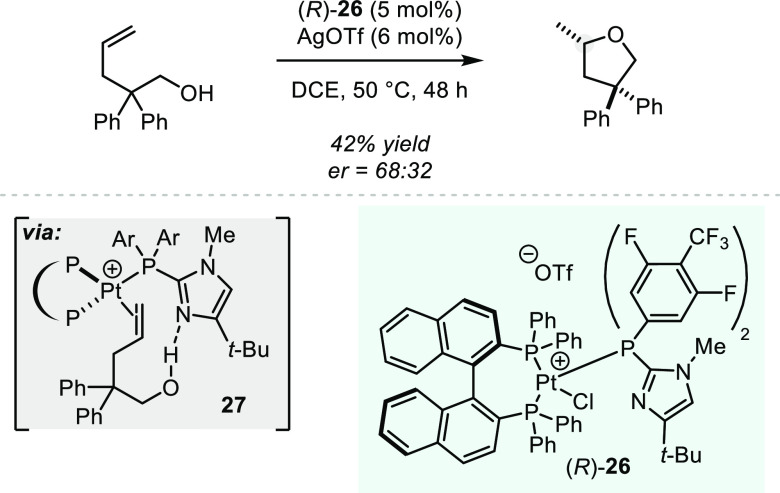
Platinum-catalyzed Asymmetric Intramolecular Hydroalkoxylation of Alkenes

In another report showcasing the ability of platinum to activate C–C double bonds, Xu and co-workers recently described a platinum(II)-catalyzed intramolecular hydroalkoxylation of alkenes (Scheme 19).^82^ The author’s approach relies on the design a of “donor–acceptor” type bifunctional Pt(II)-catalyst, where the monodentate phosphine ligand contains a basic imidazole unit that can engage in hydrogen bonding with the nucleophilic alcohol to form a well-defined TS **27**. Thus, with additional aid of a Thorpe–Ingold effect in the substrate, a chiral tetrahydrofuran was obtained in moderate yield, albeit with poor enantioselectivity. Interestingly, removal of the basic functionality in the catalyst lead to complete erosion of enantioselectivity, providing some proof for the mechanistic hypothesis. However, it should be noted that the reaction conditions employed by the authors (AgOTf in DCE at 50 °C) have been demonstrated by Hintermann to lead to the formation of TfOH via decomposition of the solvent (see Scheme 2). Although the authors describe a set of control experiments to rule out hidden acid catalysis, we believe the results are not conclusive and a more rigorous analysis would be adequate. While the observed enantioinduction does indeed imply the active involvement of platinum complex in the hydroalkoxylation, due to the low level of selectivity, nonasymmetric background reactivity cannot fully be ruled out. An additional role of the basic functionality in the catalyst might be the capture and deactivation of small amounts of triflic acid rather than the active involvement in the stereodetermining cyclization step.

**Scheme 20:**
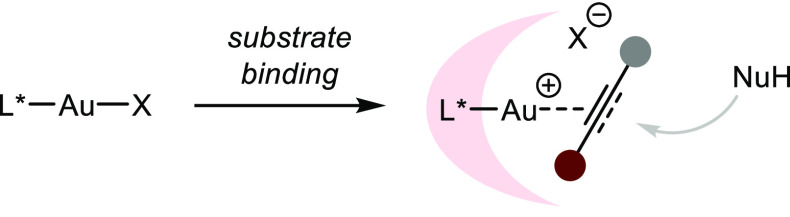
Coordination Sphere of Au(I) Complexes and the Associated Difficult Stereoinduction by the Ligand

Lewis acidic gold complexes have been widely employed in hydrofunctionalizations of allenes and alkynes. Some important features in Au(I) catalysts that define their reactivity include: (a) the propensity to coordinate to C–C multiple bonds with a strong kinetic preference to react with alkynes, (b) a well-defined and modular catalyst structure with a strong metal–ligand bond, (c) “aurophilic” behavior, i.e., a stabilizing Au–Au interaction with the magnitude of a hydrogen bond, and (d) the linear bidentate coordination geometry of Au(I) complexes (which consequently mandates outer-sphere attack) (Scheme 20).^83^ Thus, a chiral ligand is placed opposite to the outer-sphere approach of a nucleophile, rendering enantiocontrol especially difficult. Additionally, the reactivity of Au(I)-catalysts is largely defined by the properties of the corresponding counteranion, with noncoordinating ions dissociating faster from the metal center and thus facilitating substrate binding. As a general observation, Au-catalyzed asymmetric transformations have been most successful with chiral binuclear catalysts of the type L(AuX)_2_. Additionally, the prominent strategy to in situ activate Au precatalysts by chloride abstraction using silver salts introduces a second transition metal in the reaction mixture, leading to potential oligomerization and loss of the well-defined catalyst structure due to the well-known *silver effect*.^84^

**Scheme 21:**
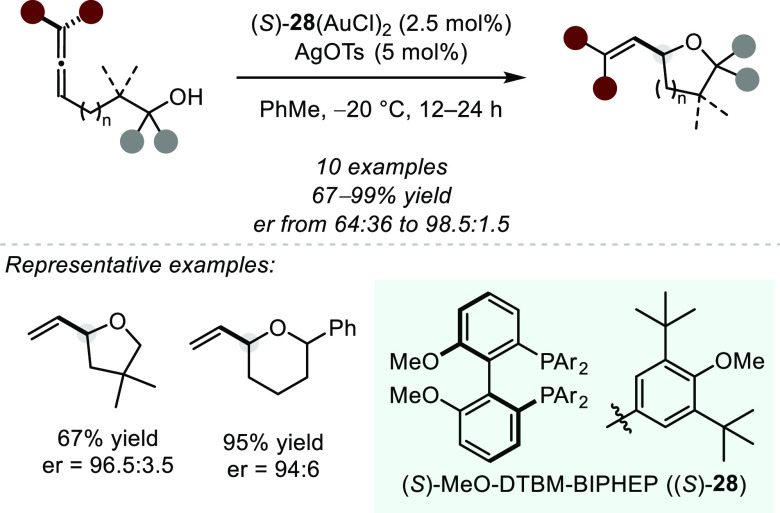
First Gold(I)-catalyzed Asymmetric Intramolecular Hydroalkoxylation of Allenes

Seminal contributions to ligand controlled asymmetric Au catalysis were made by the Widenhoefer et al., who documented the first enantioselective hydroalkoxylation of allenes catalyzed by a dinuclear Au(I) complex, formed upon activation of precatalyst (*S*)-**28**(AuCl)_2_ with AgOTs (Scheme 21).^85^ The transformation gives rise to chiral THFs and THPs in good to excellent yield and moderate to excellent selectivity; however, a significant drop in enantioselectivity was observed when employing a substrate bearing no substituents along the tether. Control experiments were conducted using a chiral AgI–phosphine complex or a chiral phosphonium salt to rule out potential background catalysis. Similar asymmetric transformations have since been reported, although no high enantioselectivity was observed for the examined ligand systems.^86,87^

**Scheme 22:**
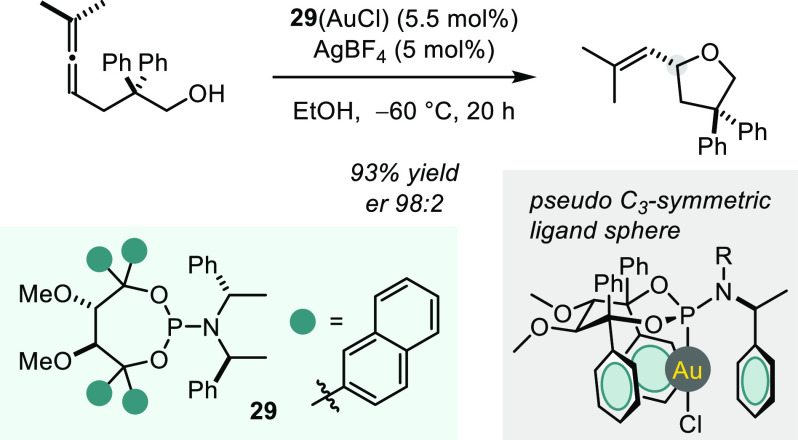
Phosphoramidite Gold(I)-catalyzed Asymmetric Intramolecular Hydroalkoxylation of Allenes

To move away from dinuclear Au complexes, Fürstner et al. designed and synthesized a series of TADDOL-related phosphoramidite ligands, one of which was successfully employed in an asymmetric cyclization of a hydroxyallene compound to a substituted tetrahydrofuran in excellent yield and enantioselectivity (Scheme 22).^88^ Notably, the authors observe a pseudo *C*_3_ symmetric ligand sphere around the Au(I) cation in the solid state of the catalyst. It was rationalized that an extended arene would allow the ligand by virtue of attractive π interactions to reach to the opposite site of the metal center and thus enable enantioinduction in an outer-sphere nucleophilic attack. A striking feature of these reactions is that the sense of asymmetric induction in the cyclization can be inverted solely by changing the solvent *or* the temperature *or* the escorting counteranion (e.g., from BF_4_^–^ to CF_3_CO_2_^–^). To gain insights into this intriguing behavior, the authors subsequently conducted experimental as well as computational mechanistic studies.^89^ Key findings include an apparent change of the stereodetermining step at reduced temperatures as well as a strong entropic contribution to the ΔΔ*G*^⧧^ of the diastereomeric transition states, leading to pronounced temperature dependency. Additionally, the proton-transfer and protodeauration steps seem to be highly dependent on the choice of solvent and counteranion, with protic solvents and coordinating ions (CF_3_CO_2_^–^) serving as proton shuttles. Remarkably, this study sheds light on the importance of entropic changes along the reaction coordinate that remain underappreciated in asymmetric catalysis.

**Scheme 23:**
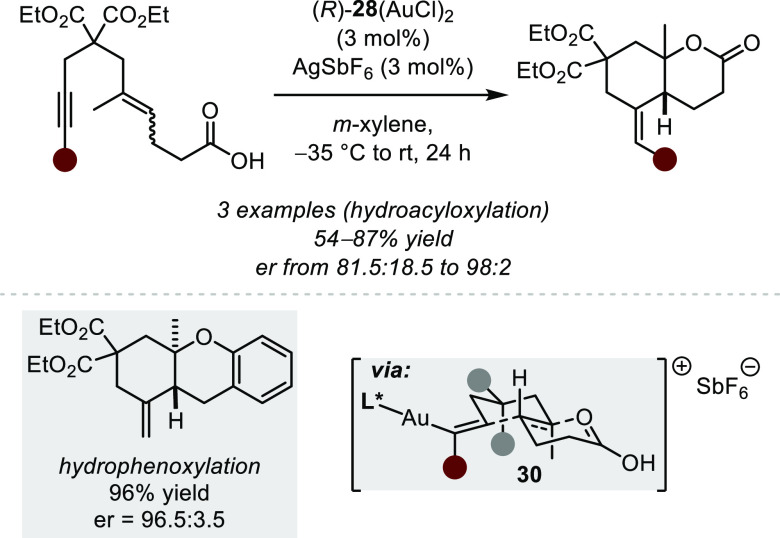
Gold(I)-catalyzed Asymmetric Polyene Cyclizations

In an impressive display of the ability of chiral phosphine–gold(I) complexes to activate alkynes, Toste and co-workers reported in 2010 enantioselective polyene cyclization cascade reactions, leading to overall hydrophenoxylations and hydroacyloxylations (Scheme 23).^90^ While analogous reactions involving the initial activation of an alkene were reported, the initiation of an asymmetric polyene cyclization by Lewis acid activation of an alkyne had remained elusive. The authors postulate that the coordination of the chiral biphenylphosphine-based Au(I) catalyst to the triple bond initiates a *6-exo-dig* cyclization, while enantioselectivity is translated by preorganization of the polyene throughout the whole cyclization cascade according to the Stork–Eschenmoser postulate (via **30**).^91^ Consequently, *cis*-fused decaline systems could also be obtained from the corresponding (*Z*)-alkenes, albeit with reduced enantioselectivity. The reaction conditions are suitable for terminal as well as internal alkynes (even though higher temperature and longer reaction times are necessary for the latter). Impressively, high enantioselectivity was not only obtained in bicyclization reactions but also in the homologated tricyclization process toward tetracyclic scaffolds.

**Scheme 24:**
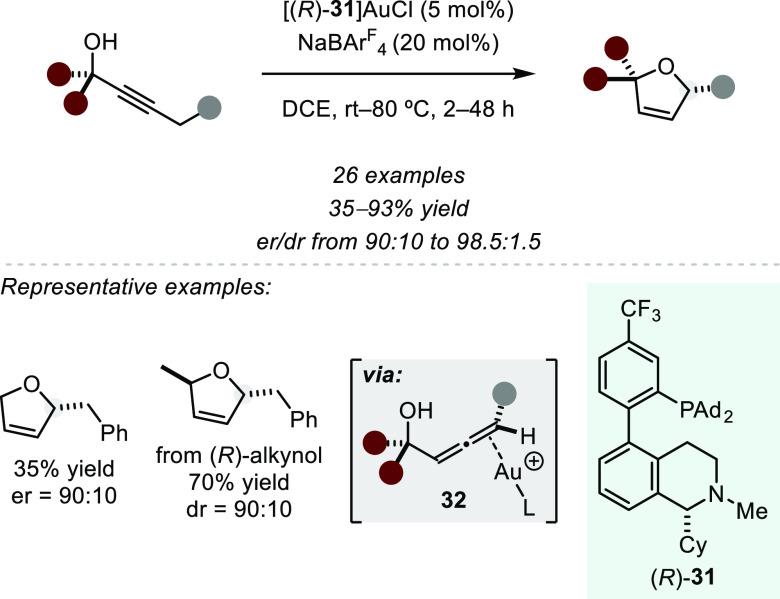
Gold(I)-catalyzed Asymmetric Alkyne Isomerization and Intramolecular Hydroalkoxylation

A rationally designed bifunctional chiral Au(I) complex was developed by Zhang et al. in 2019 and utilized in an asymmetric isomerization of an alkyne to the corresponding chiral allene and a subsequent stereospecific intramolecular hydroalkoxylation to yield 2,5-dihydrofurans in good yield and stereoselectivity (Scheme 24).^92^ Importantly, starting from enantiopure secondary alcohols, the diastereomeric ratio in the cyclization product was controlled by the catalyst and not by the substrate. To achieve efficient asymmetric isomerization, the authors deliberately introduced a basic tertiary amine into the ligand that deprotonates the substrate in the propargylic position upon Lewis acid activation by the gold complex. Interestingly, while the precatalyst [(*R*)-**31**]AuCl exists as a mixture of atropisomers that were isolated and analyzed by single crystal structure analysis, anion exchange to a noncoordinating tetraarylboronate at elevated temperatures lead to quantitative equilibration of the complex to a single isomer with (*R*_a_,*R*)-configuration. With this in situ generated catalyst isomer, the authors developed a stereochemical model where a *syn*-periplanar Au(I)-assisted deprotonation and subsequent protodeauration lead to enantioenriched Au complex **32**, from which stereospecific cyclization provides the desired product.

#### 2.2.2 Asymmetric Counteranion Directed Catalysis

In the previously discussed section, neutral chiral ligands coordinate tightly to the metal, and the stereoselectivity is induced during the interaction of these complexes with the substrates. However, complexes with cationic metal centers are necessarily associated with anions or anionic ligands. When those anions are chiral and enantiomerically pure, the corresponding reactions can be broadly classified as asymmetric counteranion directed catalysis (ACDC). In this context, chiral anions, for example, BINOL derived phosphates, in addition to neutralizing charge or acting as a (weak) ligand, are capable of engaging in interactions such as hydrogen bonding and deprotonation. There exists a continuum of possibilities, where selectivity is dependent with varying extent on electrostatic, noncovalent, and covalent interactions. A detailed discussion of ACDC is beyond the scope of this review, and interested readers are advised to consult the literature.^93,94^

**Scheme 25:**
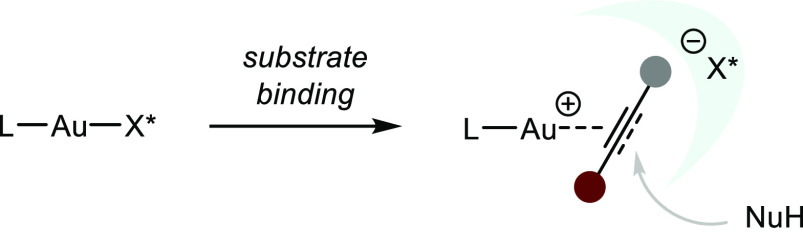
Coordination Sphere of Au(I) Complexes under the Influence of a Chiral Anion

The area of gold catalysis has offered a fertile breeding ground for the development of ACDC, as often the use of traditional ligand scaffolds result in poor selectivity due to the linear coordination geometry around the metal center (Scheme 20). On the other hand, the counteranion in Au(I) catalysis necessarily needs to dissociate from the metal center to allow substrate coordination. Thus, it is possible to create a chiral ion pair with the metal-bound substrate and guide nucleophilic approach from outside the ligand sphere (Scheme 25). It has moreover been observed that a chiral counteranion can be combined additively with a chiral ligand to enable asymmetric transformations. In this section, we will focus on examples where π-Lewis acidic metals are employed in the context of asymmetric counteranion directed hydroalkoxylation. A separate section related to ACDC within the realm of chiral Brønsted acids can be found in section 5.

**Scheme 26:**
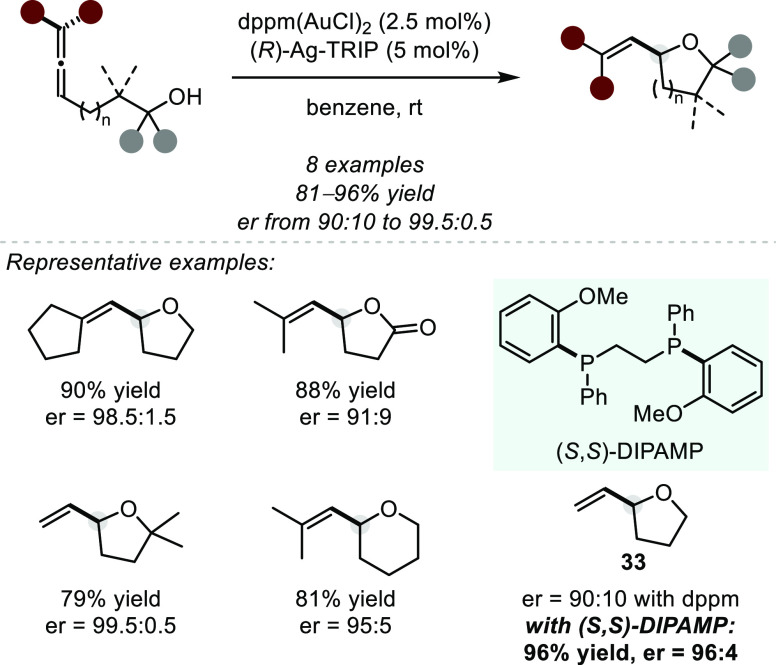
Gold(I)-catalyzed Asymmetric Hydroalkoxylation of Allenes via ACDC

Pioneering synthetic method development toward asymmetric chiral counteranion directed hydroalkoxylation can be attributed to Toste and co-workers (Scheme 26).^95^ Their first example of an intramolecular asymmetric hydroalkoxylation of an allenol can be perceived as further development of the seminal chiral ligand-controlled contribution by Widenhoefer et al. (Scheme 21). The authors found that a Au precatalyst with an achiral ligand scaffold (dppm) could be employed in the presence of (*R*)-TRIP as a chiral counteranion to effect high levels of enantioinduction in intramolecular cyclizations. The method was found to be compatible with a variety of allenol substrates, including α- or β-substituted alcohols of varying tether linkage. Notably, the synergistic nature of the ligand scaffold and chiral counteranion can be appreciated in the cyclization to form THF **33**. In this case, the anion alone induces 80% ee (er = 90:10) when the reaction is performed with dppm; however, when employing a chiral-at-phosphorus ligand (DIPAMP) together with the (*R*)-TRIP counteranion, enantioinduction is improved to 92% ee (er = 96:4). Control experiments rule out the possibility of background catalysis by the AgTRIP-salt or the corresponding CPA alone. Consistent with an ion-pair model, it was observed that nonpolar solvents gave optimal enantioselectivity while more coordinating solvents lead to erosion of selectivity due to attenuation of crucial electrostatic effects (solvent-separated ion pair). Furthermore, the authors were able to extend the concept to asymmetric hydroacyloxylations of allenes by employing (*S*)-BINAP as a chiral ligand and (*R*)-TRIP as a chiral counteranion. These reactions exhibit a strong matched–mismatched effect: When (*R*)-Ag-TRIP is combined with the antipodal (*R*)-BINAP(AuCl)_2_, the product is formed in a near-racemic fashion. Such behavior has previously also been described in organocatalytic reactions involving ion pair catalysts consisting of chiral ammonium cations with chiral phosphate counteranions.^96^ Further, this methodology was successfully applied to intramolecular asymmetric hydroamination and has since also been demonstrated in intramolecular asymmetric aminoxylations of allenes.^97^ Additionally, hydroxyl-allenecycloisomerizations have been reported under ACDC using a slightly different catalytic system.^98^

**Scheme 27:**
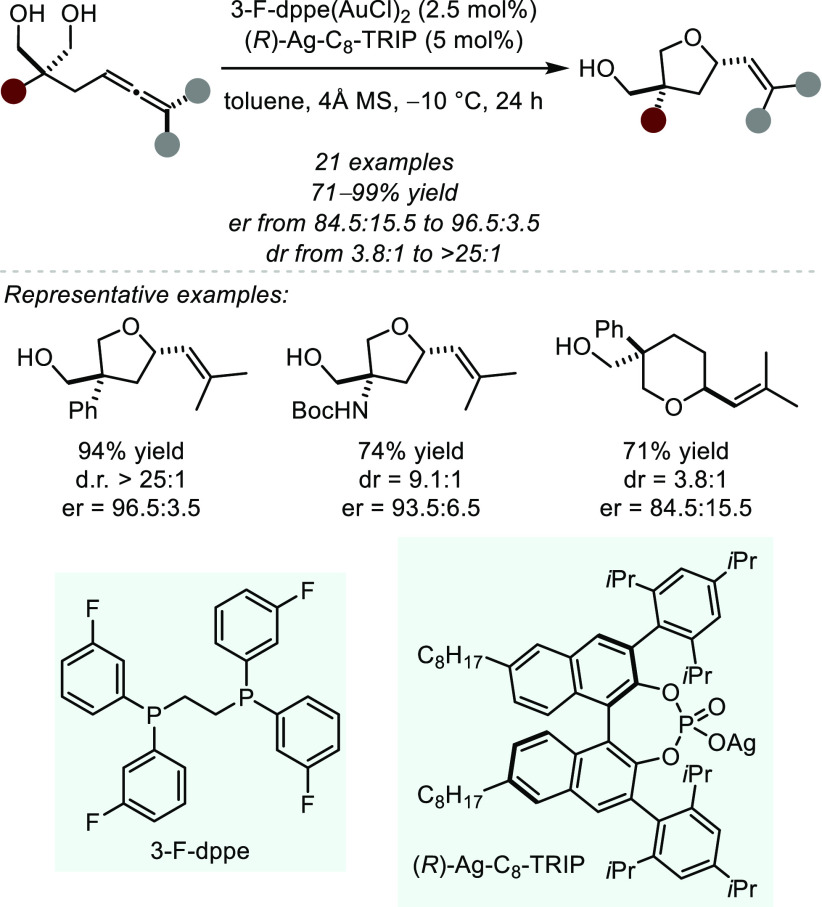
Gold(I)-catalyzed Asymmetric Desymmetrizing Hydroalkoxylation of Allene-Containing Diols

A desymmetrization of allene-containing 1,3-diols has been contributed by Toste and co-workers using a purely asymmetric counteranion directed protocol with an achiral dppe-ligand and (*R*)-Ag-C_8_-TRIP (Scheme 27).^99^ Under optimized reaction conditions, tetrahydrofurans were formed in good yield and with high selectivity. However, the homologated tetrahydropyrans displayed reduced enantioselectivity. Mechanistically, the authors did not observe a nonlinear correlation between the silver salt’s ee and the product ee, suggesting the influence of only a single chiral counteranion in the enantiodetermining step. The structure of the active dinuclear catalyst therefore remains of interest, especially with regard to the ongoing aspiration to design more efficient catalyst scaffolds.

**Scheme 28:**
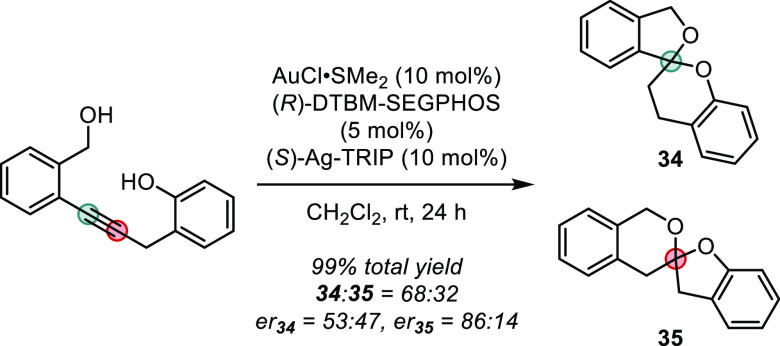
Gold(I)-catalyzed Asymmetric Dihydroalkoxylation of Alkynes toward Spiroacetals

Brimble and co-workers have leveraged a chiral counterion-based strategy to achieve an asymmetric dihydroalkoxylation of suitably crafted alkyne-diols toward valuable spiroacetals (Scheme 28).^100^ Under the influence of a Au(I)-catalyst possessing both a chiral bisphospine ligand as well as a chiral phosphate anion, separable regioisomeric mixture of spiroacetals (**34** and **35**) were obtained in quantitative yield but with poor regioisomeric ratio and moderate enantioselectivity. The intermediacy of a putative enol ether has been verified by a control experiment, in which subjecting the separately prepared intermediate to the reaction condition leads to the experimentally observed product with identical selectivity. A later example by Rexit and Mailikezati adopted a modified approach to successfully catalyze an asymmetric dihydroxylation to yield spiroacetals in good yields and enantioselectivities.^101^ As will be discussed in section 5.1, highly enantioselective spiroacetalizations have been achieved under the influence of confined organic Brønsted acid catalysts.^102^

**Scheme 29:**
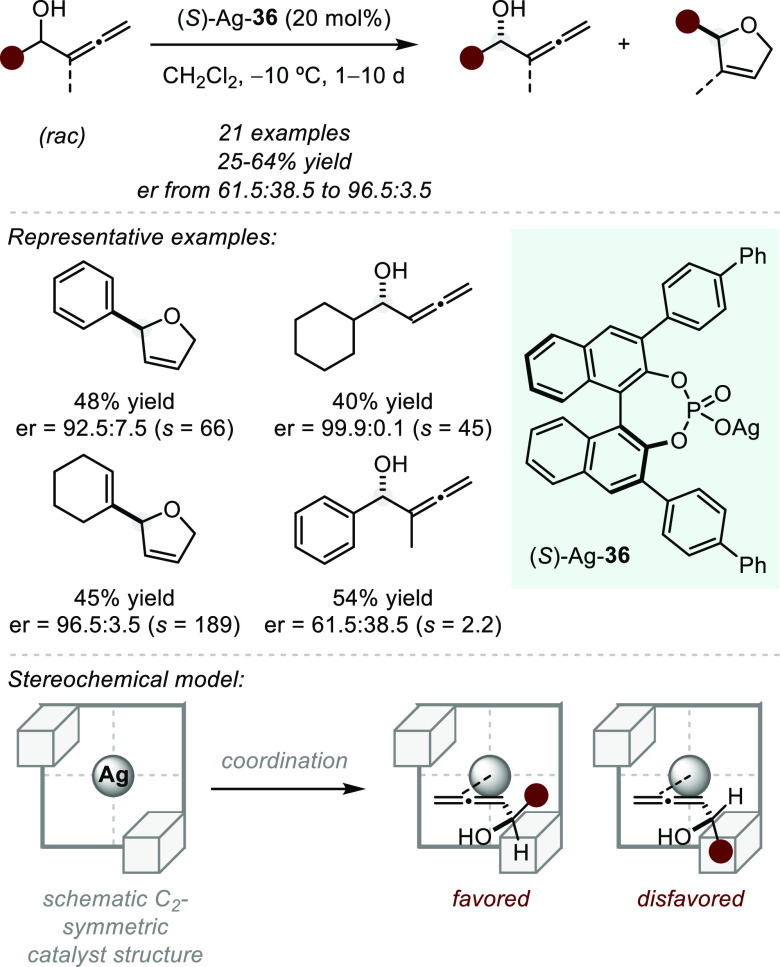
Silver(I)-catalyzed Kinetic Resolution of Allenic Alcohols

While it is very prominent within Au(I) catalysis, the use of chiral counteranions to induce enantioselectivity has also been extended to other π-Lewis acidic metals. We will conclude this section by highlighting some of these examples. Whereas chiral silver-phosphates play a crucial role in generating an active Au(I) catalyst, Ag(I) complexes can also display catalytic activity themselves. In 2012, Hong and co-workers reported a chiral silver(I)-catalyzed kinetic resolution of racemic allenic secondary alcohols toward the cycloisomerized products (Scheme 29).^103^ Using a BINOL-derived silver phosphate, good selectivity (*s* up to 189) was observed for a wide range of substrates, although high catalyst loading was required. To rationalize the observed stereochemical outcome, the authors propose a geometric model in which the allene coordinates to the silver to form an η^2^ complex. The different stability of the diastereomeric complexes formed upon complexation of either enantiomer of the starting material might account for the cyclization rate difference required for the kinetic resolution. On the basis of the steric contour of the four schematic quadrants created by the chiral phosphate anion, the (*R*)-enantiomer of the starting material will preferentially coordinate to the metal, as the sterically demanding substituent will be able to reside in the unhindered area. Additional support for this model arises from the observation that both tertiary as well as C_2_-substituted allenic alcohols cyclize with drastically reduced selectivities, presumably due to steric clash of the additional substituent with the phosphate anion. An additional mechanistic possibility includes hydrogen bonding of the phosphate anion to the free OH to increase its nucleophilicity and create a well-defined transition state.

**Scheme 30:**
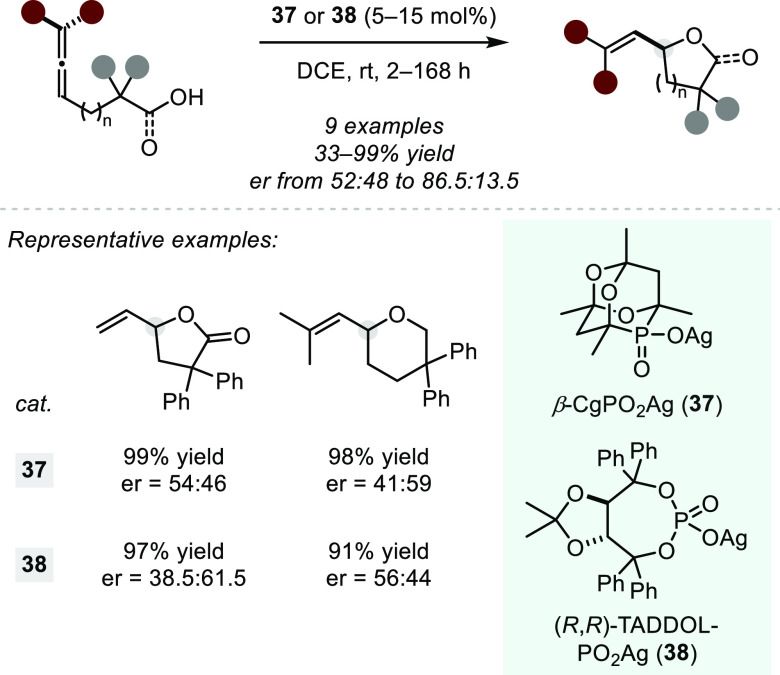
Silver(I)-catalyzed Hydroacyloxylations and Hydroalkoxylations of Allenes

In 2012, Hii and co-workers disclosed an asymmetric silver(I)-catalyzed intramolecular hydroalkoxylation and hydroacyloxylation of allenes (Scheme 30) under the influence of chiral phosphate anions.^104^ Using the silver salts of 6-phospha-2,4,8-trioxa-adamantane (β-CgPO_2_Ag, **37**) or TADDOL derived phosphoric acid ((*R*,*R*)-TADDOL-PO_2_Ag, **38**), the authors were able to obtain cycloisomerized products in quantitative yield but only with poor enantioselectivity. Additionally, the protocol relies on the use of geminal disubstituted starting materials, a setback that was already disentangled by Au(I) catalysis (Scheme 26).

**Scheme 31:**
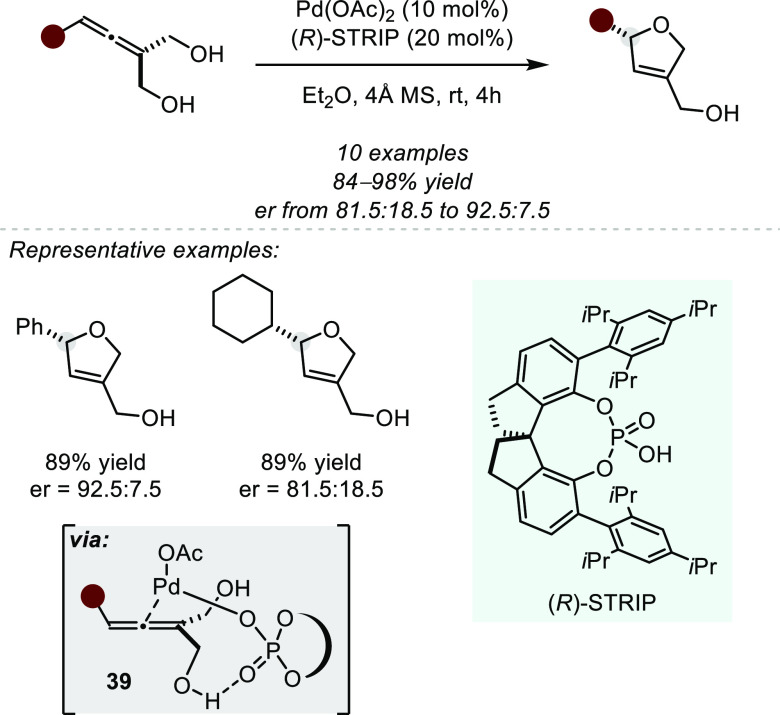
Palladium(II)-CPA-cocatalyzed Asymmetric Desymmetrizing Hydroalkoxylation of Allenic Diols

Similar to what was already discussed within the realm of Au(I) catalysis, a desymmetrization of allenic 1,3-diols has been disclosed by Cao and Zheng via Pd(II) and CPA cocatalysis.^105^ Under the influence of catalytic amounts of Pd(OAc)_2_ and SPINOL-derived phosphoric acid (*R*)-STRIP, substituted dihydrofurans could be obtained in excellent yields and moderate enantioselectivities (Scheme 31). On the basis of literature precedence, the authors propose that the phosphate serves as both an anionic ligand to the metal center and as the base to simultaneously activate one of the alcohols, thus inducing stereoselectivity (see complex **39**).

## 3 Metal-Hydride Hydrogen Atom Transfer

**Scheme 32:**
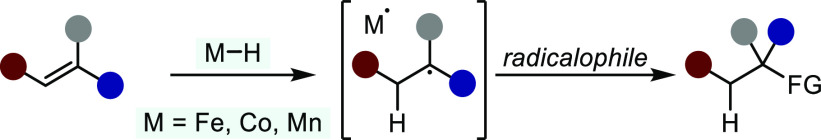
MHAT Hydrofunctionalization of an Alkene

An emerging strategy in asymmetric hydrofunctionalization chemistry capitalizes on transition-metal hydrides (M–H) that react with alkenes via hydrogen atom transfer (HAT).^106−109^ The ensuing formation of a solvent-caged metallo/organic radical pair offers a versatile platform for functionalization with a variety of coupling partners (Scheme 32). Such catalytic systems are common with first-row transition metals, including Fe, Co, and Mn, and proceed under mild reaction conditions with high levels of chemoselectivity. Additionally, the intermediacy of carbon centered radicals dictates the regioselectivity of MHAT hydrofunctionalizations and leads to Markovnikov adducts (detailed mechanistic discussion follows). The robustness of this methodology has manifested in its use in a myriad of natural product syntheses, with the Mukayaima hydration being a model example.^106^ However, because of the inherent challenge of controlling the facial differentiation of putative alkyl radical intermediates, stereoselective processes have largely been limited to auxiliary-controlled reactions.^110,111^ Only in the past few years have highly enantioselective MHAT reactions been developed, with asymmetric hydroalkoxylation reactions being among the first examples in the field.

**Scheme 33:**
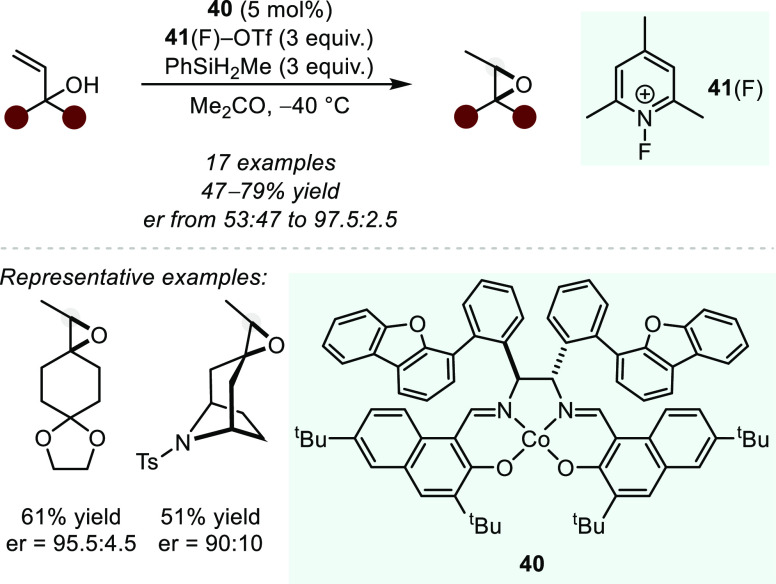
Co-catalyzed Intramolecular Hydroalkoxylation of Allylic Alcohols to Enantioenriched Epoxides

Namely, in 2019, Pronin and co-workers reported the first example of a highly asymmetric HAT-initiated hydrofunctionalization, demonstrating an intramolecular cyclization of tertiary allylic alcohols to furnish the corresponding epoxides with good to excellent levels of enantioselectivity (Scheme 33).^112^ The reaction employs methylphenylsilane as reductant and an *N*-fluoropyridinium oxidant (**41**(F)) and is catalyzed by chiral Co salen complex **40** containing dibenzofuran units bound to the ethylenediamine-derived fragment, which prove to play an crucial role in enantioinduction (vide infra). High levels of asymmetric induction were observed with heterocycle-derived allylic alcohols (e.g., tetrahydropyrans or piperidine derivatives) as well as cyclohexanes bearing heteroatoms. Alternatively, substrates derived from simple cyclohexanes, including those containing alkyl substituents, resulted in decreased enantioselectivities. Further, employment of an acyclic substrate yields the desired epoxide, however, with nearly complete loss of enantioinduction.

**Scheme 34:**
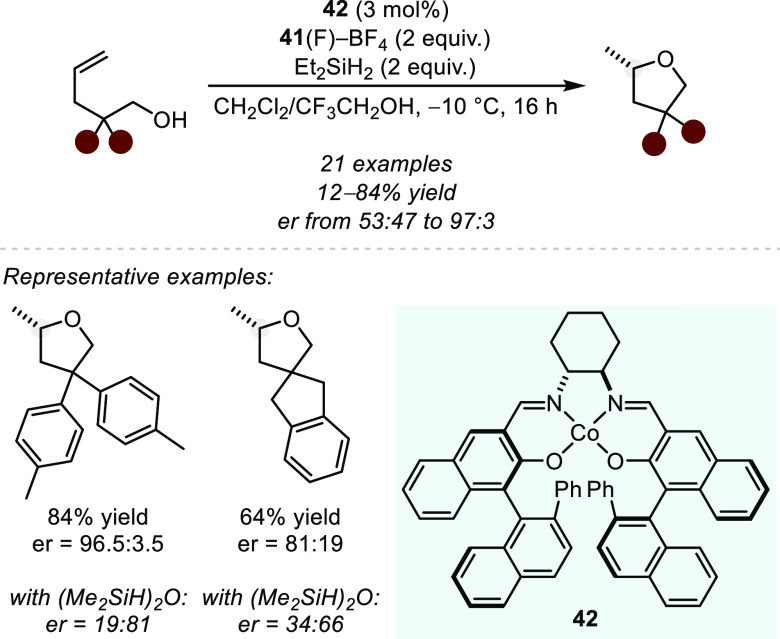
Co-catalyzed Intramolecular Asymmetric Hydroalkoxylation toward Tetrahydrofurans

Alternatively, in 2016, Shigehisa and co-workers reported a single example of an intramolecular asymmetric hydroalkoxylation of 2,2-diphenylpent-4-en-1-ol to yield the corresponding tetrahydrofuran with moderate enantioselectivity (er = 64:36).^113^ In 2020, the same research group showed that incorporation of chiral binaphthyl units into the Co salen scaffold dramatically improves the observed enantioinduction (Scheme 34).^114^ The authors demonstrated a moderate scope of alkenols, however, all substrates contain bulky substituents along the tether. Surprisingly, the steric nature of the employed silane has a striking effect on the enantioselectivity of the reaction. In particular, relatively nonsterically hindered secondary silanes, e.g., diethylsilane, selectively form the (*S*)-enantiomer of the product. Alternatively, more sterically encumbered silanes, e.g., diisopropylsilane and tetramethyldisiloxane, result in the formation of the product antipode.

**Scheme 35:**
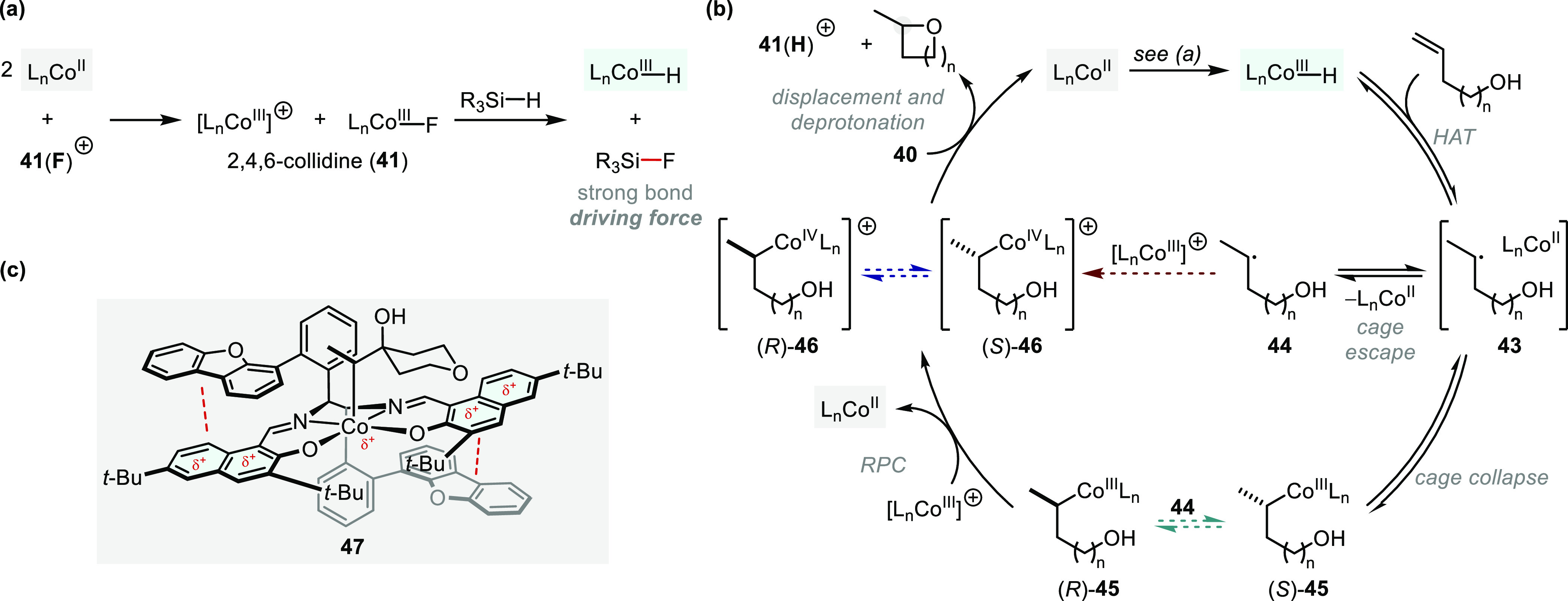
A General Mechanistic Proposal for Asymmetric MHAT Hydroalkoxylation

A general mechanistic proposal for Co-catalyzed HAT-initiated hydroalkoxylation is depicted in Scheme 35. While it is broadly accepted that such transformations proceed through metal hydrides, the mechanism to form the requisite M–H is not yet well understood, particularly with Co salen complexes. Shigeshi and Hiroya have suggested that the catalytic cycle commences with the oxidation of two equivalents of a Co(II) precatalyst with an *N*-fluoropyridinium salt (**41**(F)) to concomitantly form a cationic Co(III) complex and a Co(III)–F intermediate (as well as 2,4,6-collidine).^113,115^ The Co(III)–F species subsequently reacts with a silane (R_3_Si–H) via ligand exchange to furnish the catalytically active Co(III)–H species, thermodynamically driven by the formation of a strong (and chemically inert) Si–F bond (Scheme 35a). However, in a recent mechanistic investigation into a Co/Ni dual-catalyzed hydroarylation, Shenvi et al. disclosed that the reaction of a Co(II) salen complex with an *N*-fluoropyridinium triflate does not lead to the isolation of a Co(III)–F complex but rather exclusive isolation of a cationic Co(III) species with an outer-sphere triflate counteranion.^116^ The authors suggest that Co–H formation could thereafter result from hydride delivery from a pentavalent silicate intermediate to the cationic Co(III) species, still leading to the formation of a Si–F bond.^108^

While detailed investigations are still necessary to disentangle the mechanistic possibilities toward M–H formation with Co salen complexes, a key feature undoubtedly includes oxidation to Co(III), as the Co(II) precatalyst is inactive in MHAT reactions.^116^ Further, a compatible reductant (typically a silane) is required and the uphill formation of a highly reactive (and weak) M–H bond must be compensated by the concomitant formation of a strong bond (typically a Si–F or Si–O bond).^108^ This represents the enabling driving force of MHAT catalysis. Recent methods, such as those described herein, employ *N*-fluoro salts, as these are not only capable oxidants but further provide the fluoride required for Si–F bond formation.

In the presence of an alkene, HAT from the transient Co–H forms a metal radical/carbon radical cage pair (Scheme 35b, **43**).^117,118^ Subsequent reactivity can proceed via three pathways: (1) a hydrogen atom abstraction by the metal can occur to reform the starting materials (and can also lead to alkene isomerization^119^), (2) the radical pair can undergo solvent cage escape, generating a free alkyl radical (**44**), or (3) the radical pair can collapse to form a diastereomeric mixture of the corresponding organometallic intermediate (**45**).^116,120,121^ In an earlier example of a nonasymmetric intermolecular hydroalkoxylation, Shigehisa and Hiroya proposed that a free alkyl radical escapes from the cage and is subsequently oxidized to form a carbocation, which is then captured by an alcohol.^115^ However, this scenario cannot explain the enantioselectivity observed in the presence of chiral Co–salen complexes. Alternatively, in 2018, Pronin and co-workers reported a catalyst-controlled chemodivergent functionalization of tertiary allylic alcohols to yield either the above-mentioned epoxides (with low ee in the model system using a chiral Co–salen complex not optimized for enantioinduction) or semipinacol rearrangement products.^122^ The striking influence of the salen scaffold on both the chemoselectivity, as well as the diastereoselectivity of the semipinacol rearrangements, led Pronin et al. to suggest that these reactions involve alkyl–Co(IV) complexes as electrophilic intermediates, rather than free carbocations. In particular, the authors propose a radical polar crossover (RPC) process in which complex **45** undergoes a single electron oxidization to furnish a reactive cationic alkyl–Co(IV)intermediate **46**, thus enabling a stereoinvertive nucleophilic displacement. Concomitant deprotonation yields the corresponding epoxide. This elementary step regenerates the Co(II) precatalyst, necessitating the stoichiometric use of oxidant and silane. These postulations are supported by pioneering work by Halpern and others describing the formation^123−128^ and ensuing reactivity^129−131^ of alkyl–Co(IV) complexes.

In their 2019 report (Scheme 33), Pronin et al. propose that the observed enantioselectivity may result from reversible epimerization of the stereocenter bearing the homolytically labile Co–C bond in **46** (blue dashed arrows), followed by an enantiodetermining displacement via a dynamic kinetic resolution. Eyring analysis using sterically and electronically differentiated salen complexes shows that the enantioselectivity of this transformation is enthalpically controlled and positively correlates to the polarizability and quadrupole moment of the arene ring in the backbone of the salen complex. Hence, the authors suggest that cation−π interactions between the radical cation of the Co(IV)–salen complex, and the electron-rich dibenzofuran motifs play an integral role in stabilizing the transition state, leading to the major enantiomer (Scheme 35c, **47**).^132^ Alternatively, the authors do not rule out the possibility of an enantiodetermining radical capture by a cationic Co(III) complex to give a scalemic mixture of the cationic Co(IV) intermediate (maroon dashed arrow). In either scenario, a stereospecific displacement of (*S*)-**46** predominates and renders the formation of the observed (*R*)-epoxide.

The profound effect of the employed silane on the observed enantioselectivity in the asymmetric hydroalkoxylation reported by Shigehisa et al. indicates that at least two competing enantiodetermining steps are operating. The authors propose that, in the presence of sterically unencumbered secondary silanes, an extensive radical chain reaction between **45** and a diffused radical (**44**) results in a diastereomeric enrichment of (*R*)-**45** (teal dashed arrows). The subsequent oxidation would therefore furnish a scalemic mixture of **46**, favoring (*R*)-**46**, from which stereospecific displacement yields enantioenriched (*S*)-product. On the other hand, when employing sterically bulky silanes, the authors suggest that this radical chain reaction is suppressed and propose a dynamic kinetic resolution analogous to Pronin to yield the (*R*)-enantiomer. The authors provide computational evidence for favorable CH−π interactions between the catalyst and substrate that stabilize either the favored alkyl–Co(III) intermediate or the transition state of nucleophilic displacement.

Given that the mechanistic scenarios for asymmetric induction involve, to varying extents, cationic intermediates in the enantiodetermining step, it might be interesting to study the effects of chiral anions on the enantioselectivity of these reactions. To the best of our knowledge, chiral anions have not yet been employed in MHAT reactions. Further, while an intermolecular asymmetric hydroamination has been recently described,^133^ intermolecular MHAT hydroalkoxylation reactions have so far not been rendered asymmetric. In a recent perspective, Holland and Shenvi et al. suggest that analogous differentiation of Fe- or Mn-based diastereomeric organometallic intermediates may be untenable; however, the authors offer other potential approaches to asymmetric MHAT method development.^108^

## 4 Photocatalysis

Photocatalysis has emerged as a powerful tool used to enable new activation modes and offer complementary reactivity to thermally catalyzed methodologies. Similar to MHAT strategies, photocatalysis offers a mild and highly chemoselective approach to the generation of reactive radical intermediates. However, in contrast to MHAT, photocatalytic hydrofunctionalization methods provide linear adducts, a notable aspect because anti-Markovnikov products are otherwise challenging to obtain. As mentioned in section 3, the intermediacy of highly reactive radical intermediates presents challenges in inducing enantioselectivity, and further, nonstereoselective background reactivity can hamper light-driven processes. Nevertheless, two general approaches to enantioselective photocatalysis have been developed. Type I describes the excitation of a substrate within a preassembled chiral environment, whereas in type II, the enantioselective bond formation is catalyzed by a “conventional” chiral catalyst and occurs separately from the photochemical reaction (this strategy is mainly used in photoredox catalysis).^134−136^ This section will include a detailed discussion of the photocatalytic methods developed for asymmetric hydroalkoxylation and will consider future directions within this subfield.

**Scheme 36:**
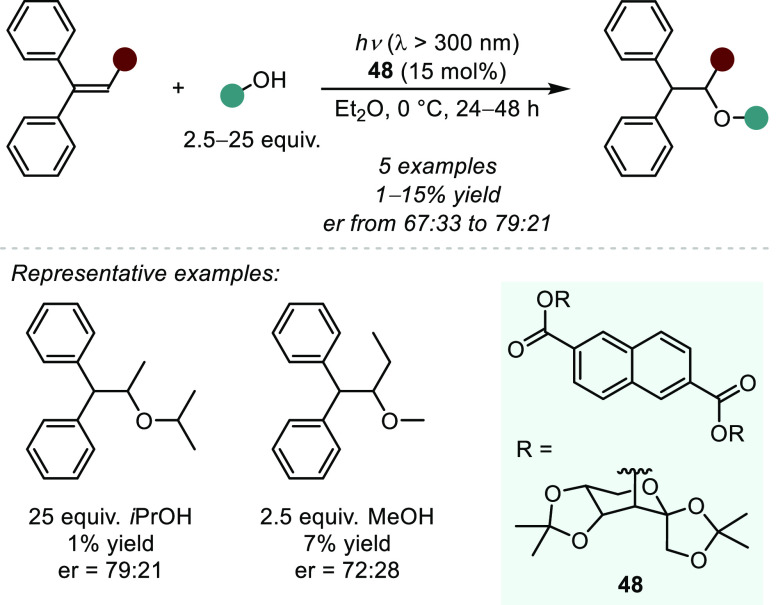
Chiral Photosensitizer-catalyzed Intermolecular Hydroalkoxylation of Stilbene Derivatives

**Scheme 37:**
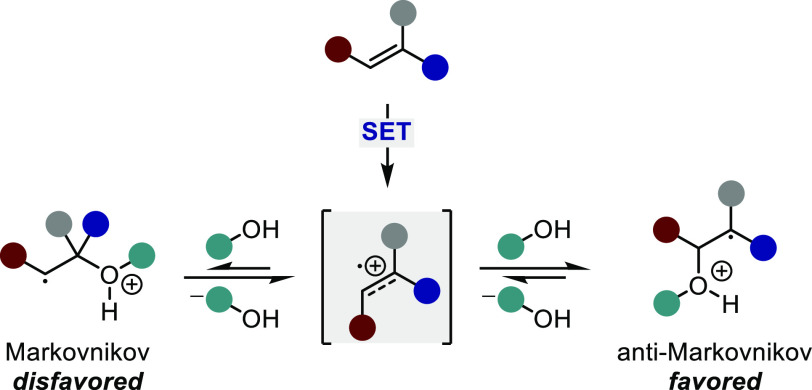
General Regioselectivity Observed for the Addition of Nucleophiles to Olefin Radical Cations

One of the earliest reports of an enantioselective intermolecular hydroalkoxylation was disclosed in 1993 by the Inoue group and describes a photocatalytic anti-Markovnikov hydroalkoxylation of stilbene derivatives.^137^ In a later development, naphthalene dicarboxylate catalyst **48** containing chiral saccharide-based motifs was employed to enable improved enantioselectivities (Scheme 36).^218^ The authors propose that an arene exciplex is formed between the catalyst and alkene substrate and that the polar nature of the auxiliary units facilitates electron transfer from the alkene to furnish a chiral radical ion pair, of which dissociation is disfavored in relatively nonpolar reaction media (Et_2_O). Hence, the olefin radical cation is contained within a chiral environment, and facial differentiation can therefore be achieved upon addition of an alcohol (type I). The regioselective preference is dictated by the favored nucleophilic attack to the less-substituted carbon of the olefin cation radical, ultimately giving rise to the anti-Markovnikov adduct (Scheme 37).^138^ While moderate levels of enantioselectivity are observed, the poor reactivity of this method restricts its overall applicability.

**Scheme 38:**
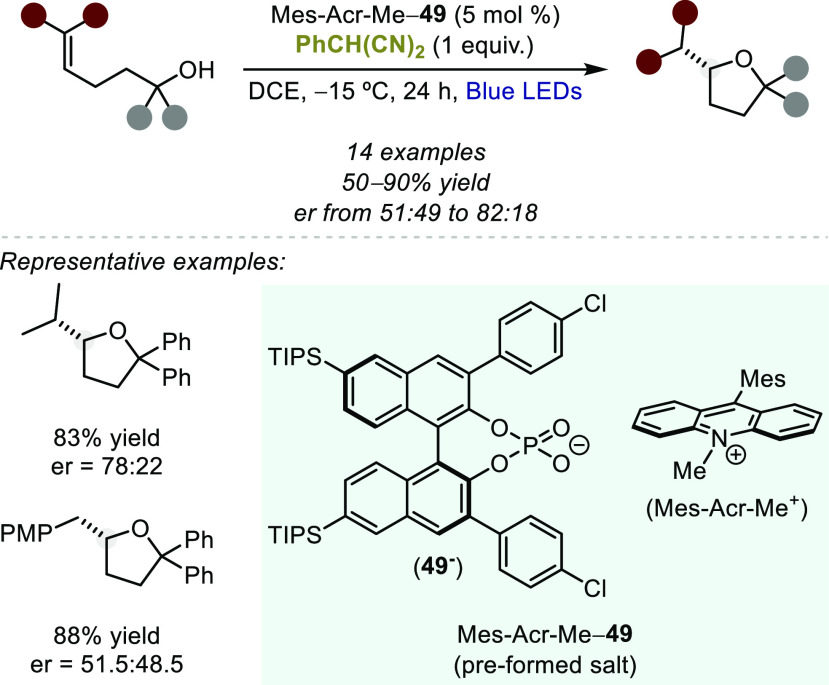
Asymmetric Hydroalkoxylation of Alkenols Using Acridinum-based Photocatalyst

Since 2012, the Nicewicz group has published a range of reports on anti-Markovnikov selective hydrofunctionalizations of alkenes using an acridinium-derived photocatalyst^139^ in the presence of a hydrogen atom transfer reagent.^140^ As with the previous example, these reactions proceed through the intermediacy of an olefin radical cation and are therefore highly selective toward linear products. Nicewicz and Hamilton initially demonstrated the success of this methodology with an intramolecular hydroalkoxylation reaction of alkenols to form a wide range of tetrahydrofurans and pyranes using 2-phenylmalononitrile as an H atom donor.^141^ In 2017, Luo and co-workers recognized that the cationic nature of the photocatalyst, as well as the intermediacy of cationic species in this transformation, revealed an opportunity to induce enantioselectivity via ion-pairing catalysis, specifically asymmetric counteranion directed catalysis. Indeed, the authors demonstrated that in the presence of a chiral ion pair photoredox organocatalyst, derived from Mes-Acr-Me^+^ and a chiral BINOL-based phosphate anion (**49**^–^), moderate levels of enantioselectivity could be achieved (Scheme 38).^142^

**Scheme 39:**
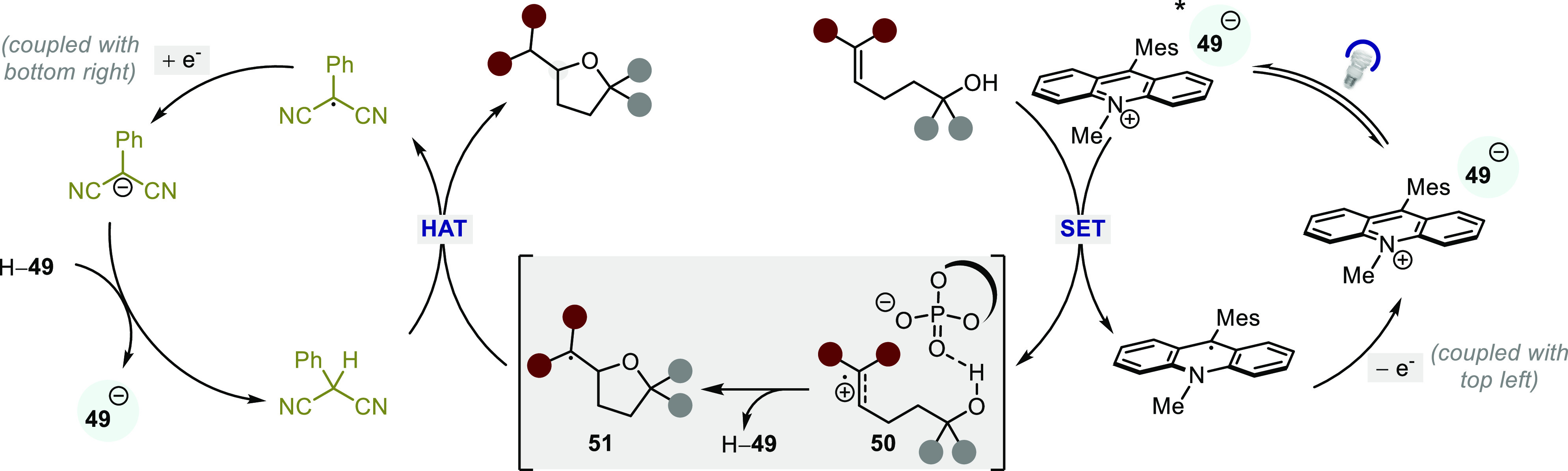
Proposed Mechanism for the Intramolecular Hydroalkoxylation with an Acridiunium-based Photocatalyst and an HAT Reagent in the Presence of a Chiral Phosphate Anion

In alignment with thorough mechanistic studies performed by Nicewicz and Romero,^143^ the authors propose a catalytic cycle entailing a photoinduced SET from the alkenol to the excited Mes-Acr-Me–**49** to yield radical cation **50**, followed by an enantiodetermining C–O bond-forming cyclization/proton transfer step to provide **51** (type II). Finally, a hydrogen atom transfer event regenerates the chiral anion and furnishes the desired product (Scheme 39). Further mechanistic studies by Luo et al. revealed that the chiral phosphate anion plays three critical roles in the catalytic process: (1) to increase the lifetime of the chiral photocatalyst’s triplet state, (2) to induce asymmetry in the cyclization step, and (3) to facilitate the proton shuttle in the cyclization/proton transfer process through H-bonding. Given the poor to moderate enantioselectivities observed, coupled with the known effect of confined organocatalysts (see section 5.1), the use of imidodiphosphate anions could potentially render this reaction highly enantioselective. Additionally, while Nicewicz has demonstrated intermolecular reactivity with methanol, so far this strategy has not been extended to an intermolecular asymmetric hydroalkoxylation and is still limited to alkenols benefiting from the Thorpe–Ingold effect.

**Scheme 40:**
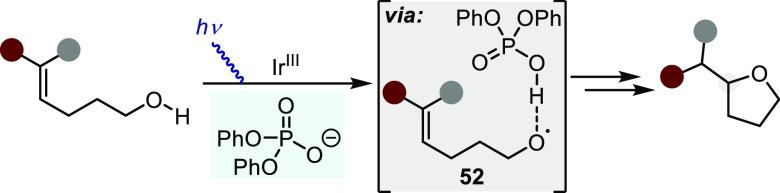
Hydroalkoxylation via Proton-coupled Electron Transfer (PCET) Activation of Alcohol O–H Bond

Knowles and co-workers recently reported a fundamentally unique approach to photocatalyzed hydroalkoxylation reactions, in which homolytic activation of an O–H bond initiates an intramolecular addition to variously substituted alkenes (Scheme 40).^144^ In particular, a phosphate anion promotes a proton-coupled electron transfer (PCET) activation of the O–H bond in the presence of an Ir^III^-based visible light photo-oxidant and subsequently mediates the ring-closing event via complex **52**. This work has, however, not yet been rendered asymmetric.

## 5 Bro̷nsted Acid Catalysis

**Scheme 41:**
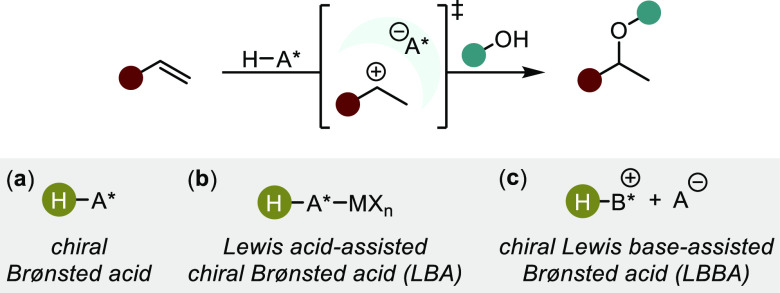
General Approaches to Brønsted Acid-catalyzed Hydroalkoxylation

Inspired by the impeccable enantioselectivities observed in Brønsted acid–mediated hydroalkoxylations using enzymes (section 7), coupled with the challenges of hidden acid catalysis in transition metal systems (section 2), chiral small molecule Brønsted acid catalysis stands out as a promising approach to developing asymmetric hydroalkoxylation methods. The formation of the branched (i.e., Markovnikov) adduct of acid-mediated hydrofunctionalizations with aliphatic alkenes is both kinetically and thermodynamically favored, and therefore such reactions typically proceed with high levels of regioselectivity.^1^ However, there exists a number of important challenges associated with this approach to asymmetric hydroalkoxylation and hydroacyloxylation. Namely, alkenes are weakly basic^145^ functionalities and therefore protonating such motifs could require very strong acids and/or harsh reaction conditions, both of which might lead to issues with functional group tolerance. Further, controlling the facial selectivity of nucleophilic attack onto intermittent carbocations is notoriously challenging^146,147^ and moreover, controlling such intermediates via ion-pairing is not well-explored. Nevertheless, a few small-molecule acids have effected moderate to excellent levels of enantioinduction in hydroalkoxylations of C–C multiple bonds. These systems can be divided into three general categories: (a) chiral Brønsted acid catalysis, (b) Lewis acid-assisted chiral Brønsted acid (LBA) catalysis, and (c) chiral Lewis base-assisted Brønsted acid (LBBA) catalysis (Scheme 41). This section will briefly introduce each of these strategies and specifically focus on their applications in asymmetric hydroalkoxylation. Similar to discussion in section 2.2.2, for gold-catalyzed reactions proceeding through ACDC, there exists a continuum of noncovalent interactions that dictate stereoselectivity, i.e., ion pairing and/or hydrogen bonding. When relevant and elucidated by the authors, such distinctions will be made.

### 5.1 Chiral Brønsted Acid Organocatalysis

Since seminal reports in 2004 by Akiyama^148^ and Terada^149,150^ on Mannich-type reactions, BINOL-based chiral phosphoric acids (CPAs) and their derivatives have proven to be privileged motifs in asymmetric catalysis.^151−153^ In sections 2.2.2 and 4, we delineated the effective use of chiral phosphates (e.g., TRIP) as anions in asymmetric transformations of C–C multiple bonds, in which either a metal or photocatalyst activates the substrate and the chiral phosphate provides enantioinduction through ion pairing with cationic intermediates. Alternatively, the direct use of CPAs as chiral Brønsted acids to protonate C–C multiple bonds and subsequently control facial selectivity of nucleophilic attack on carbocation intermediates presents an elegant approach to asymmetric hydroalkoxylation. However, the relatively weakly acidic nature of CPAs (p*K*_a_ = 12.5–14 in MeCN^154^) is inherently limiting and has rendered aliphatic alkenes out of reach. Strategies to overcome this limitation have involved the use of electronically activated alkenes (e.g., enol ethers) or, more recently, the design of very strong and confined Brønsted acids that are capable of protonating unactivated alkenes and imparting stereocontrol on corresponding carbocation intermediates. This section will provide an overview of each of these approaches.

**Scheme 42:**
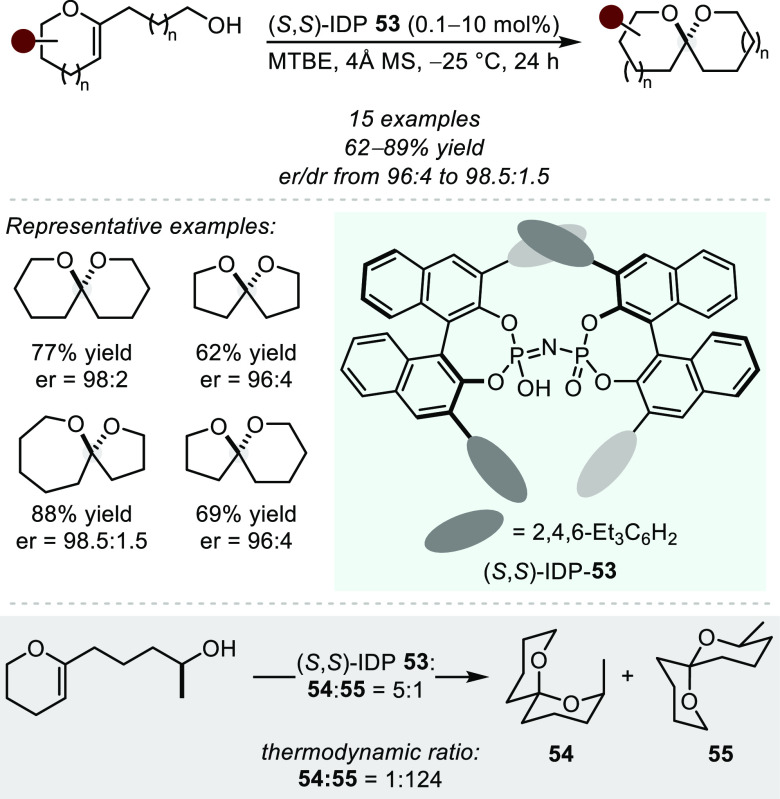
IDP-catalyzed Hydroalkoxylation of Enol Ethers toward Enantioenriched Spiroacetals

Upon the basis of our group’s previous endeavors in asymmetric spiroacetalizations,^155^ in 2012, we described a catalytic enantioselective spiroacetalization reaction of hydroxyenol ethers (Scheme 42).^102^ The success of this method was enabled by the development of a new class of highly confined *C*_2_-symmetric imidodiphosphates (IDPs), which maintain the bifunctional nature of CPAs while providing an enzyme-inspired geometrically constrained reaction site (p*K*_a_ = 11.5 in MeCN,^156^ when 3,3′ substituents = −C_6_H_5_). The employed IDP catalyst **53** is tolerant of a range of substrates to yield the corresponding spiroacetals with low catalyst loading (as low as 0.1 mol %), including in a highly enantioselective synthesis of (*S*)-olean, a sex pheromone of female olive fruit flies. Impressively, with chiral nonracemic hydroxyenol ether starting materials, the catalyst displays exquisite stereocontrol, overriding the thermodynamic preference of the substrate and providing a range of nonthermodynamic spiroacetals with good levels of diastereoselectivity.

**Scheme 43:**
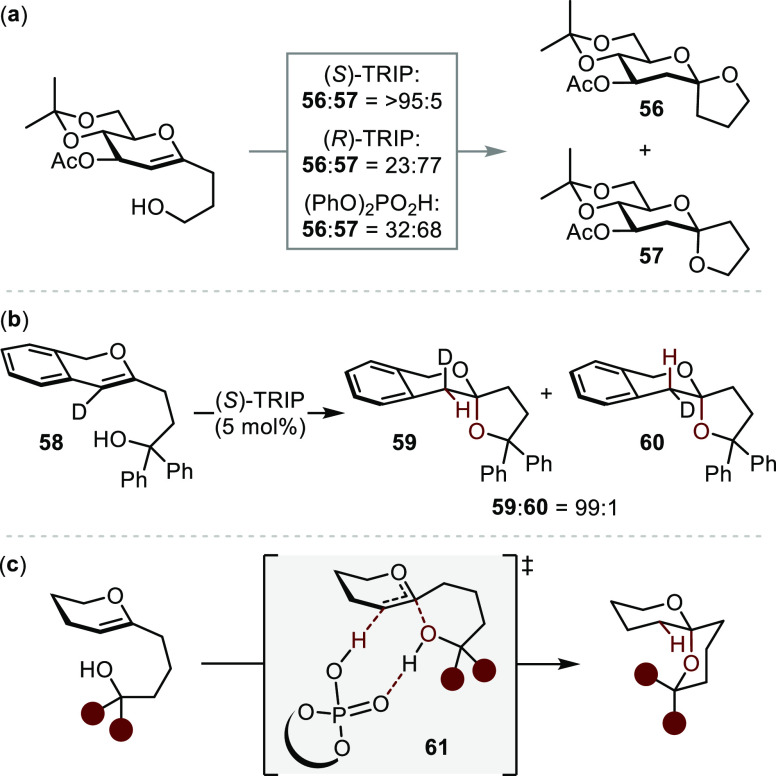
(*S*)-TRIP-catalyzed Asymmetric Hydroalkoxylation of Enol Ethers

Soon after, the Nagorny group published a conceptually similar approach with a highly stereoselective spiroacetalization using (*S*)-TRIP.^157^ The substrates employed do show dependence on Thorpe–Ingold substituents along the tether, perhaps a consequence of the more open active site of CPA catalysts in comparison to the IDP catalyst employed in the above example from our group. Alternatively, the authors additionally employed a range of d-glycal derivatives and observed nonthermodynamic spiroacetals in high yields and good to excellent diastereoselectivities (Scheme 43a).

Accompanied mechanistic and computational studies were conducted in order to differentiate between potential S_N_1-like, S_N_2-like, and covalent phosphate intermediate-based mechanisms.^158^ In particular, the authors employed deuterium-labeled hydroxyenol ether **58** and concluded that the TRIP-catalyzed spiroacetalization proceeds via a *syn*-selective addition of the O–H group across the C–C double bond (Scheme 43b). Further, a significant inverse secondary kinetic isotope effect was observed for this substrate, suggesting that rehybridization occurs in the rate-determining step (RDS). Additionally, a Hammett analysis conducted with aromatic enol ethers is consistent with the buildup of positive charge in the transition state of the RDS, although the absolute value suggests a concerted pathway rather than the formation of a fully charged oxocarbenium ion. Taken together, and in combination with in-depth computational studies (including use of the growing string method and molecular dynamics), the authors suggest that these TRIP-catalyzed spiroacetalizations occur via a concerted, though asynchronous mechanism in which a fully formed oxocarbenium ion is not implied. Instead, the bifunctional nature of the CPA is exploited to initially effect protonation of the enol ether followed by deprotonation of the alcohol appendage and simultaneous spiroacetalization via TS **61** (Scheme 43c).

While the above examples achieve reactivity with phosphoric acid-derived catalysts, the dependency on electronically activated C–C multiple bonds limits their generality in asymmetric hydroalkoxylation reactions. Alternatively, the use of electronically unbiased alkenes have eluded chiral Brønsted acid catalysis since seminal reports, and the design and synthesis of stronger BINOL-derived chiral acids has been at the center of research efforts in asymmetric organic Brønsted acid catalysis for nearly two decades. To this end, our group recently disclosed imidodiphosphorimidate (IDPi) catalysts,^159^ which not only maintain the confined reaction pocket of the above-mentioned IDP’s but additionally offer significantly increased acid strength due to Yagupolskii-type^160^ substitution of P=O double bonds with P=NTf motifs (p*K*_a_ = 4.5 in MeCN,^156^ when 3,3′ substituents = −C_6_H_5_).

**Scheme 44:**
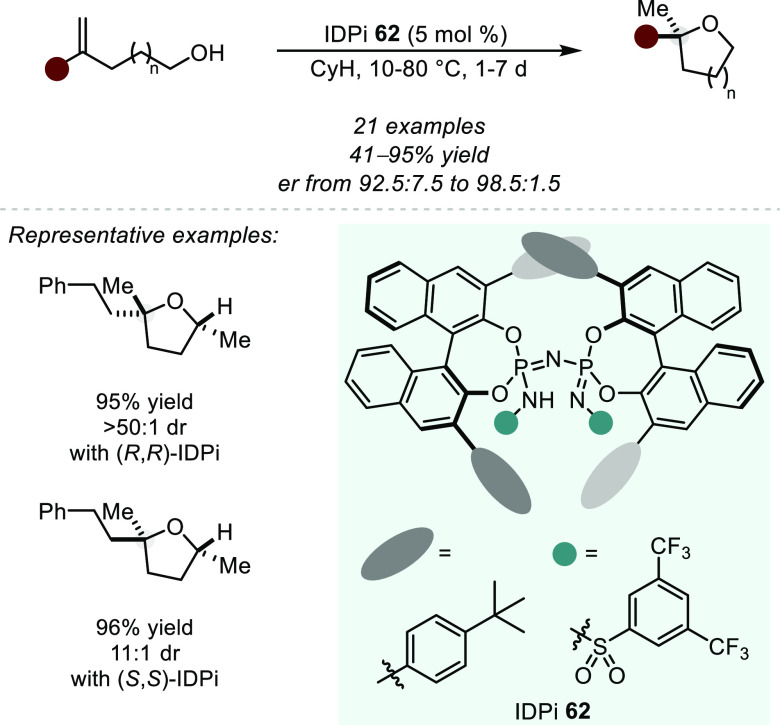
IDPi-catalyzed Asymmetric Intramolecular Hydroalkoxylation of Simple Alkenols

With such scaffolds in hand, our group disclosed an intramolecular hydroalkoxylation of simple alkenols to provide highly sought-after enantioenriched tetrahydrofurans and tetrahydropyrans in high yields (41–95%) and excellent enantioselectivities (er = 92:8 to 98.5:1.5) using IDPi **62** (Scheme 44).^17^ Importantly, opposed to the intramolecular examples described throughout section 2.1.1, this method does not require Thorpe–Ingold substituents along the tether of the alkenol to induce cyclization. In fact, a substrate bearing a dimethyl unit along the chain proved significantly less reactive, underscoring the sensitivity of the confined active site. To probe the mechanism and origin of enantioselectivity for this transformation, a combination of computational and experimental investigations were carried out. Analogous to the previous example by Nagorny and Zimmerman et al., density functional theory (DFT) studies (B3LYP/def2- TZVP/D3(BJ)/CPCM) suggest that the reaction proceeds through a concerted, though asynchronous mechanism in which the reaction is triggered by protonation of the alkene followed by enantiodetermining C–O bond formation. Further, an intramolecular competitive Hammett analysis with a series of styrene derivatives was performed. Plotting log(*k*_X_/*k*_H_) against substituent parameter σ^+^ results in a linear correlation with a negative slope (ρ= −2.08 ± 0.04), corroborating the buildup of carbocationic character at the internal position of the double bond.

**Scheme 45:**
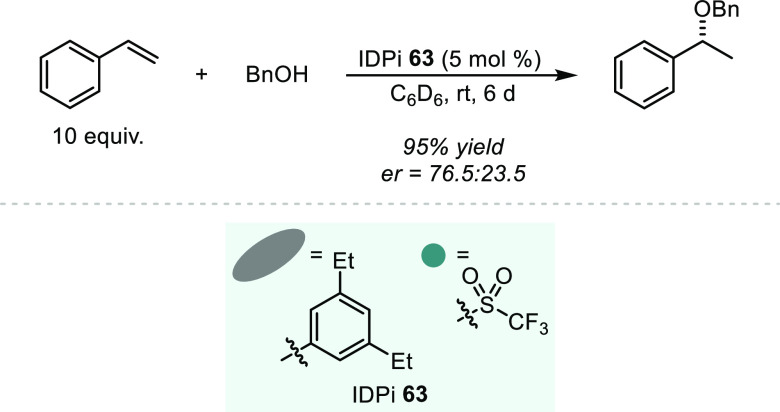
IDPi-catalyzed Asymmetric Intermolecular Hydroalkoxylation of Styrene

The generality of this method was further showcased by the encouraging enantioselectivity (er 76.5:23.5) observed during the initial screening of highly challenging intermolecular hydroalkoxylation (Scheme 45).^17^ This elegant design demonstrates the truly unique capabilities of small-molecule catalysts to exert enzyme-like sterocontrol while simultaneously opening the door to explore new reactivity that were previously beyond the reach of the traditional organocatalysts.

### 5.2 Lewis-Acid Assisted Chiral Brønsted Acid Catalysis

**Scheme 46:**
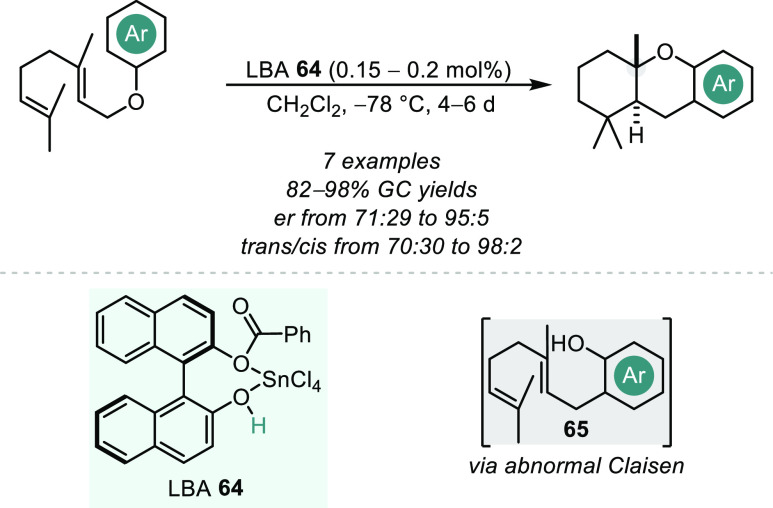
Enantioselective Cyclization of Geranyl Aryl Ethers via Abnormal Claisen

In 1994, Yamamoto and co-workers demonstrated that the combination of an achiral Lewis acid and a chiral Brønsted acid results in a conformationally rigidified chiral Brønsted acid with increased strength, termed by the authors as a Lewis acid-assisted chiral Brønsted acid (chiral LBA).^161−163^ Chiral LBAs are typically generated in situ by combining optically active binaphthyl derivatives with equal or excess amounts of Lewis acid at room temperature to form solution-stable coordinated complexes. Early studies highlighted the use of LBAs in asymmetric protonations of silyl enols ethers with the first catalytic variant reported in 1996.^164^ In 1999, Yamamoto and co-workers published a breakthrough report on small-molecule mediated asymmetric polyene cyclizations using chiral LBAs.^165,166^ Substrates containing a free hydroxy group (polyolefinic phenols and alcohols) require stoichiometric amounts of the chiral LBA, likely a consequence of the −OH group competitively binding to the Lewis acid and impeding reactivity. Alternatively, enantioselective cyclizations of geranyl aryl ethers proceed smoothly with catalytic quantities of chiral LBA **64** to provide the cyclized products with moderate enantioselectivity and high degrees of diastereoselectivity (Scheme 46). The authors suggest that this reaction proceeds through a [1,3] rearrangement (abnormal Claisen rearrangement) to provide polyolefinic phenol **65** that subsequently undergoes cyclization. On the basis of these successes, Yamamoto and co-workers published a series of reports throughout the early 2000s, demonstrating the robust ability of chiral LBAs to mediate a range polyene cyclizations en route to natural products, including syntheses of (−)-ambrox,^13^ (−)-chromazonarol,^12^ and (−)-caparrapi oxide,^167^ although these examples require stoichiometric quantities of a chiral LBA. In 2009, Bhat et al. employed chiral LBA-mediated polyene cyclizations in the total syntheses of (+)-sclareolide and (+)-tetrahydroactinidiolide, starting from (3*R*,6*E*)-nerolidol and (*R*)-linalool, respectively.^168^ In this case, carboxylic acids were used as terminating groups and the cyclizations proceeded with high levels of enantioinduction with substoichiometric amounts of the chiral BINOL derivative (in the presence of stoichiometric quantities of SnCl_4_).

**Scheme 47:**
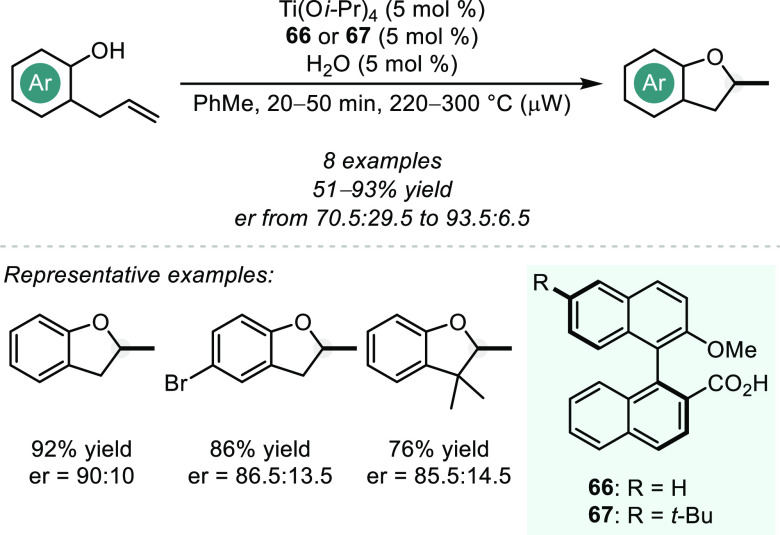
Intramolecular Hydroalkoxylation of 2-Allylphenols to 2-Methylcourmarans

More recently, in 2015, the Hintermann group showed that an in situ generated enantiopure titanium-derived complex catalyzes the cyclization of 2-allylphenols to yield enantioenriched 2-methylcourmarans (Scheme 47).^169^ The chiral catalyst is prepared by combining an enantiopure 1,1′-binaphthyl-2-carboxylic acid (BINA-Cox), Ti(Oi-Pr)_4_, and a cocatalytic quantity of water in a 1:1:1 ratio (5 mol % each). Despite requiring very high temperatures (240 °C, μW), the intramolecular cyclizations proceed with moderate enantioselectivites.

Recent efforts by the authors have focused on expanding the limited reactivity scope by preparing and testing a diverse library of BINA–Cox ligands (>30).^170^ While incremental improvements to the substrate scope were achieved, this method has so far not been successfully applied to aliphatic alkenols. The authors suggest that a multinuclear titanium-μ-oxo species is in situ generated and acts as the active catalytic species, although they have not yet confirmed the structure of the formed complex. Further, the authors have not yet proposed a mechanism for this transformation, although some insight can be gained in a related nonasymmetric hydroalkoxylation by the authors with Al(i-PrO)_3_.^171^

**Scheme 48:**
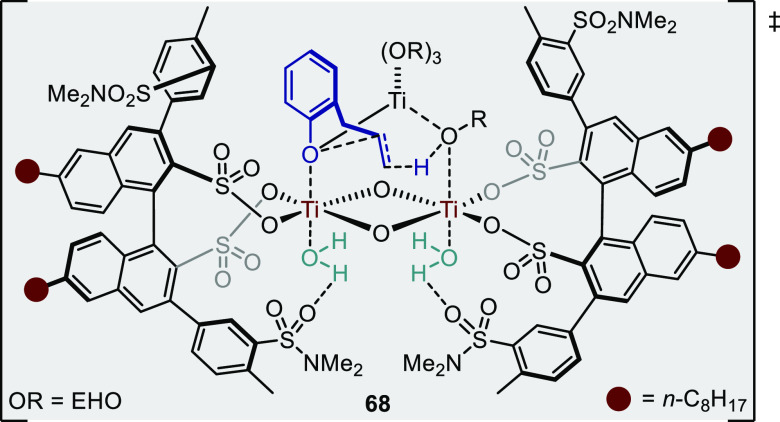
Proposed Transition State in Disulfonic Acid/Ti/H_2_O-Catalyzed Cyclization of 2-Allylphenols

In 2021, Xie and Li reported a highly related intramolecular hydroalkoxylation using a chiral 1,1′-binaphthyl-2,2′-disulfonic acid (BINSA) in the presence of Ti(EHO)_4_ (EHO = 2-ethylhexyloxy) and water.^172^ The shown intramolecular cyclizations of 2-allylphenols proceed under comparatively mild reaction conditions (75 °C) and generally improved enantioselectivities are observed, relative to the previous example. In line with Hintermann, the authors propose that such transformations proceed through a multinuclear titanium-μ-oxo species and have tentatively provided a possible mechanism, with the enantiodetermining step involving concomitant protonation of the alkene and C–O bond formation to yield enantioenriched cyclized products (Scheme 48).

**Scheme 49:**
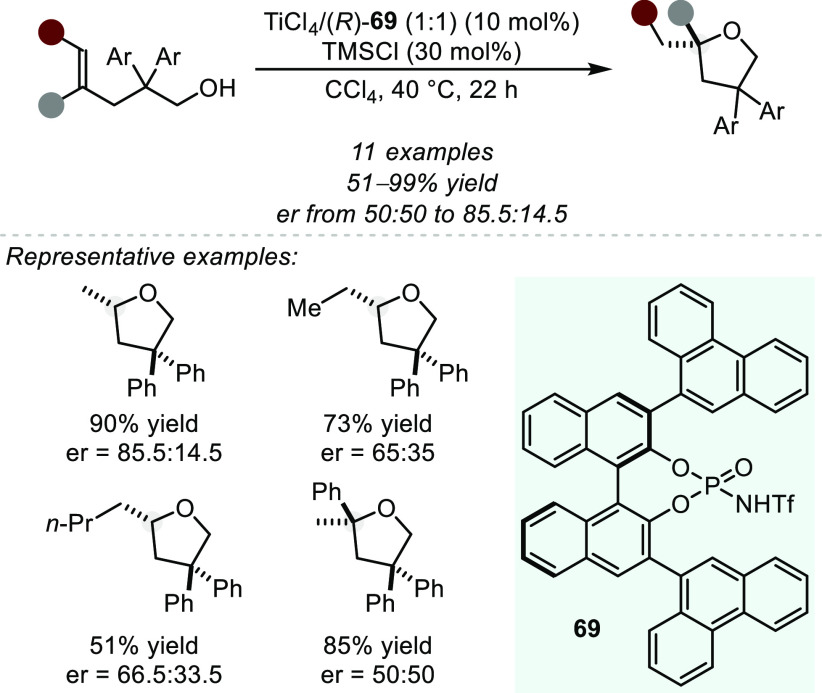
Chiral *N*-Triflyl Phosphoramide/TiCl_4_-catalyzed Intramolecular Hydroalkoxylation

In 2020, Zhao, Zhao, and co-workers capitalized on the combination of chiral *N*-Tf-phosphoramide **69** and TiCl_4_ to effect an asymmetric hydroalkoxylation of alkenols with moderate levels of enantioselectivity (Scheme 49).^173^ Similar to aforementioned examples using transition metals, the reactivity of this system is highly dependent on bulky substituents along the chain to promote substrate cyclization through the Thorpe–Ingold effect. Replacement of the aromatic rings with either methyl groups or hydrogens resulted in either a near or a complete loss of reactivity, respectively. Notably, the *N*-Tf-phosphoramide Brønsted acid cannot alone promote the cyclization, likely a consequence of inadequate Brønsted acidity. While the authors propose a structure for the Ti(VI)-(*R*)-**69** complex, there is currently insufficient evidence to distinguish whether this complex acts a chiral Lewis acid or chiral Brønsted acid. Despite this mechanistic ambiguity, it is herein categorized under LBA catalysis for consistency of reaction conditions (combination of a chiral Brønsted acid and a strong Lewis acid) and because a key step of the reaction mechanism likely includes protonation of the C–C double bond.

### 5.3 Chiral Lewis Base Assisted Brønsted Acid Catalysis

**Scheme 50:**
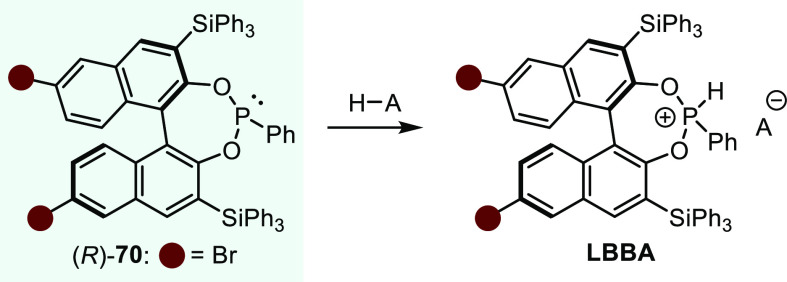
Generalized Concept of LBBAs

Complementary to previously introduced LBAs, the combination of an achiral Brønsted acid with a chiral enantiopure Lewis base is coined Lewis base–assisted Brønsted acid (LBBA) catalysis. Notably, while the design of LBAs relies on the increased Brønsted acidity upon Lewis acid activation, LBBAs show diminished acidity with regard to the original acid. Therefore, an excess of Lewis base catalyst with regard to the acid is usually required to suppress unselective background reactivity. Also, a significant difference in p*K*_a_ between Lewis base and Brønsted acid is crucial for quantitative consumption of the achiral acid. The first LBBAs introduced by Ishihara and co-workers in 2011 fulfill these criteria by incorporating a sufficiently Lewis basic chiral BINOL-derived phosphonate and a strong Brønsted acid (Scheme 50).^174^

**Scheme 51:**
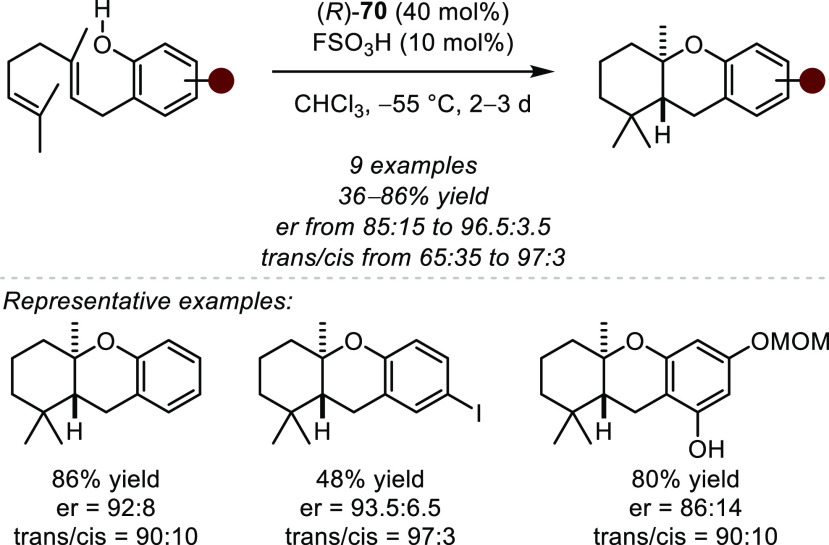
Asymmetric LBBA-catalyzed Polyene Cyclization

Chiral Lewis bases similar to **70** were previously employed in asymmetric halopolycyclizations using *N*-iodosuccinimide (NIS) by Ishihara and co-workers.^175^ Because of the intermediacy of an ionic (R_3_P-I^+^) species that acts as an electrophilic activator to induce high enantioselectivity in the cyclization, the authors speculated that the replacement of iodine by a simple proton would result in a similarly structured Brønsted acid that might be able to induce a biomimetic polyene cyclization. In their seminal report, Ishihara and co-workers thus utilized LBBAs for this purpose (Scheme 51).^174^ The authors were able to obtain trans-fused tricyclic frameworks with good yield and high diastereo- and enantioselectivity from a variety of substituted 2-geranylphenols. Importantly, sterically nondemanding Brønsted acids like fluorosulfonic acid showed generally improved enantioselectivity. Additionally, fine-tuning of the electronic and steric properties of the Lewis basic phosphine turned out to be crucial for high yield, enantioselectivity, and reduced catalyst decomposition under the reaction conditions. With a related catalytic principle, the same group in 2013 reported the LBBA-catalyzed kinetic resolution of racemic 2-substituted carboxylic acids via intramolecular hydroacyloxylation.^176^

## 6 Lewis Base Catalysis

**Scheme 52:**
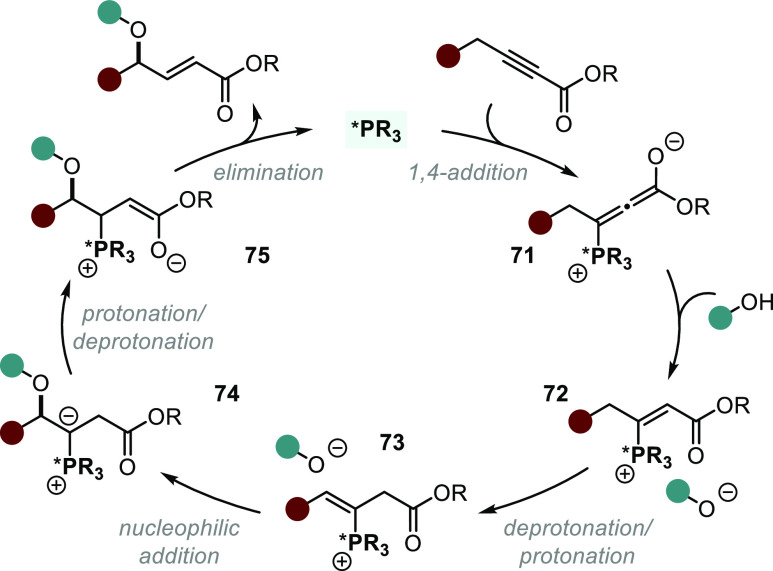
General Mechanism of Phosphine-catalyzed Hydroalkoxylations of Propiolate Derivatives

As was discussed in the previous section, chiral phosphines can act cooperatively with achiral acids to form Lewis base assisted Brønsted acid catalysts. In a distinct activation mode, phosphines can also engage with activated alkynes, related to the well-explored Morita–Baylis–Hillman reaction, as nucleophilic Lewis bases. In 1994, Trost and co-workers established that in the presence of 1,3-bis(diphenylphosphino)propane (DPPP), an intramolecular hydroalkoxylation of ω-hydro-alkynoates can be achieved.^177^ As outlined in Scheme 52, the mechanism commences with the 1,4-addition of a chiral phosphine to an alkynoate to give zwitterionic intermediate **71**. Subsequently, a series of proton transfers leads to the key electrophilic cationic intermediate **73** that, after nucleophilic addition, generates ylide intermediate **74**. Further proton transfer generates an enolate **75** that releases the catalyst upon product formation. Notably, the overall reaction can be understood as a γ-umpolung of alkynoates, with a formal oxidation in the γ-position, while the alkyne is reduced to an alkene. Akin to some π-Lewis acidic metals (see section 2), these transformations are compatible with both alkynes and allenes, as both proceed via a common catalytic intermediate.^178^

**Scheme 53:**
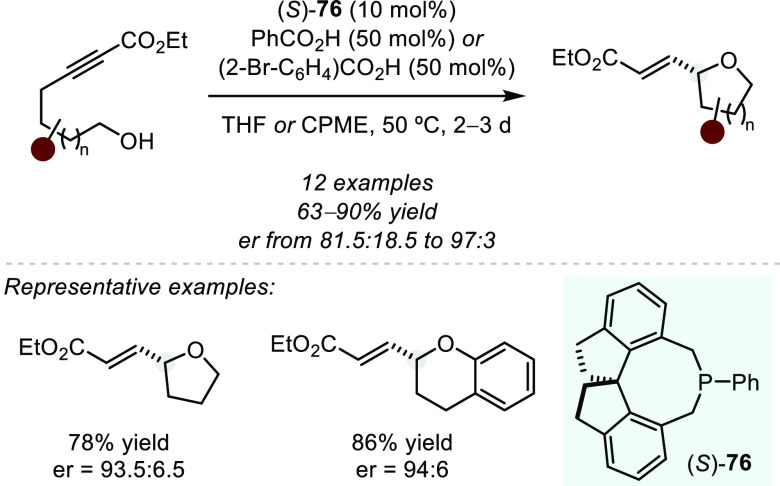
Phosphine-catalyzed Asymmetric Intramolecular Hydroalkoxylation of Alkynoates

Because of the covalent nature of the catalytic intermediates formed between the Lewis basic catalyst and the substrate, a reasonable advancement was the development of chiral phosphines for asymmetric hydroalkoxylations of alkynoates. The first asymmetric variant of this reaction was reported in 2009 by Fu and co-workers (Scheme 53).^179^ Using spiroindane-derived phosphepine **76** as catalyst, the authors achieved an asymmetric intramolecular hydroalkoxylation to furnish THFs, THPs, and (iso)chromanes in good yields and enantioselectivities. Notably, the use of benzoic acid derivatives as additives had a profound effect on reactivity and selectivity. Control experiments show that the added carboxylic acid does not protonate the phosphine. Possibly, it is instead involved in the proton transfer reactions as well as in the stereodetermining step, as an engineered bromobenzoic acid was required to achieve high enantioselectivity for phenolic substrates. Additionally, spectroscopic analysis of the reaction mixture identified the resting state of the catalyst as the free phosphine rather than a phosphonium adduct with the substrate.

**Scheme 54:**
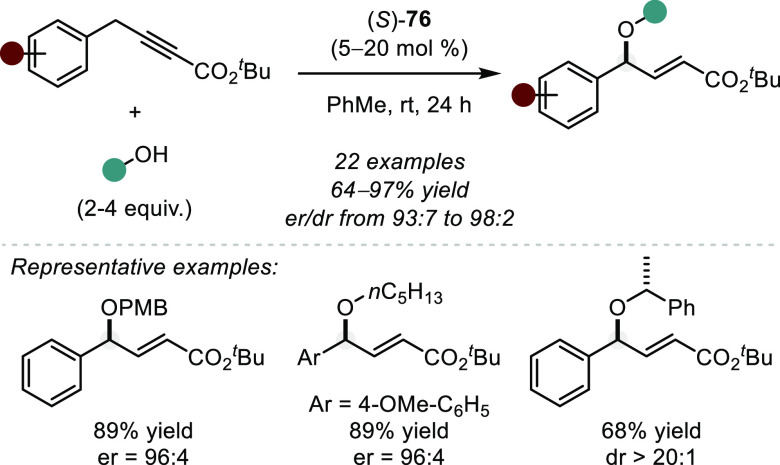
Phosphine-catalyzed Asymmetric Intermolecular Hydroalkoxylation of Alkynoates

Fu and co-workers further demonstrated the generality of phosphephine catalyst **76** in an intermolecular hydroalkoxylation of benzylic alkynoates (Scheme 54).^180^ A wide array of aromatic substrates as well as alcohols are well tolerated, furnishing the ethereal products with generally high yield and selectivity. Remarkably, the catalyst control was further highlighted in the reaction with enantiomerically pure α-methyl benzyl alcohols, where high diastereomeric control was imparted regardless of the nucleophiles absolute configuration. Despite the impressive stereochemical outcome, the transformation still suffers from several drawbacks. First, the transformation relies on aromatic substrates. Second, sterically demanding secondary alcohols like cyclohexanol are not well tolerated, giving significantly diminished product yield. Further, the utilization of water as nucleophile was impossible under the reported reaction conditions. Improvements in these directions would represent a significant leap toward general protecting group free functionalization of alkynoates.

## 7 Enzyme Catalysis

Given the vast ubiquity of hydrofurans and hydropyrans in natural products, Nature’s chemical toolkit includes several highly stereoselective methods for the formation of cyclic ethers. Known biosyntheses of these motifs include general acid- and general base-catalyzed additions of alcohols to epoxides, carbonyls, and Michael acceptors.^181^ Despite the efficiency and versatility of hydroalkoxylation processes, known enzymatic asymmetric additions of alcohols to C–C π bonds remain relatively rare, and to the best of our knowledge, are limited to intramolecular reactions. In this section, we will delineate enzyme-catalyzed asymmetric hydroalkoxylations and discuss recent advances in hydration reactions of alkenes.

**Scheme 55:**
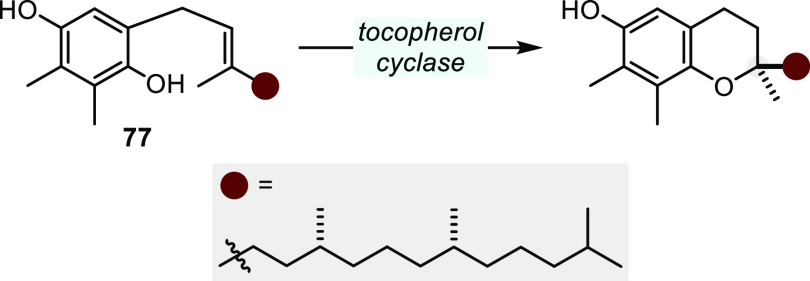
Enzyme-catalyzed Hydroalkoxylation Toward γ-Tocopherol

In the early 1990s, Woggon and co-workers identified a previously undiscovered enzyme responsible for the asymmetric cyclization of **77** to γ-tocopherol, a key step in the biosynthetic route toward the vitamin E family (Scheme 55).^182^ Labeling studies revealed that the enzyme-catalyzed cyclization proceeds through *Si*-protonation of the double bond and concomitant *Re*-attack of the phenolic oxygen to furnish γ-tocopherol.^183^ Further, the authors discovered three critical substrate-enzyme recognition sites: (1) the OH group at C(1) of the hydroquinone, (2) the (*E*)-configuration of the double bond, and (3) the length of the lipophilic side chain. Variation of these components, i.e., acylation of the alcohol, use of the (*Z*)-isomer, or shortening of the side chain, results in either significantly reduced yields or no observed cyclization.^184^ Alternatively, mimicking the stereocontrol of asymmetric cyclizations toward this chromane framework using strong chiral Brønsted acids has proven to be a formidable challenge.^185^ As such, the development of small-molecule catalysts capable of achieving such reactivity with high levels of enantioinduction and a potentially increased reactivity scope is warranted.

**Scheme 56:**
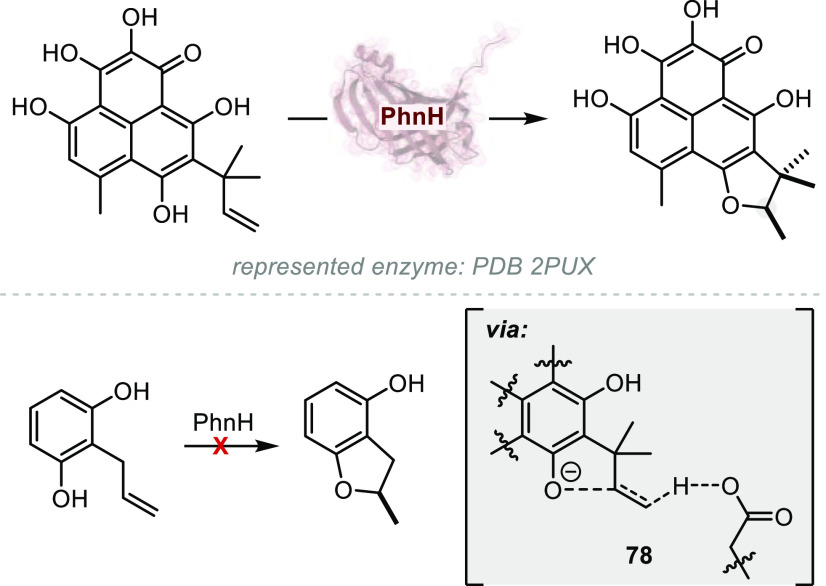
An Intramolecular Asymmetric Hydroalkoxylation in the Biosynthesis of Herqueinone

More recently, Garg, Houk, and Tang et al. identified a dedicated enzyme that catalyzes an enantioselective hydroalkoxylation reaction in the biosynthesis of herqueinone, a fungal metabolite. In particular, the authors discovered a 149aa protein encoded from the gene *PhnH* that is responsible for the intramolecular addition of a phenol to the terminal alkene of a reverse prenyl group (Scheme 56).^186^ DFT calculations (using acetic acid as a general acid model), coupled with enzymatic modeling and docking studies, suggest that the reaction is initiated by deprotonation of the phenol group, followed by C–O bond formation and a concerted proton transfer from a nearby glutamate residue (via TS **78**).^187^ Notably, the authors do not rule out an alternative mechanism in which the glutamate residue positions a water molecule as a specific acid in the protonation step. Unfortunately, attempts to translate this chemistry to a simplified and non-natural substrate, 2-allyl resorcinol, resulted in no rate acceleration of the hydroalkoxylation adduct. To our knowledge, promiscuous reactivity of engineered analogues of this enzyme with non-natural substrates has yet to be studied, but could offer meaningful solutions to the field.

While enantioselective enzymatic hydroalkoxylations are still largely undiscovered, several enzymes are known to catalyze intermolecular asymmetric hydrations of electronically unactivated C–C double bonds. For example, fatty acid hydratases convert isolated alkenes of unsaturated fatty acids to the corresponding chiral alcohols.^188^ Additionally, linalool dehydratase/isomerase catalyzes an asymmetric hydration reaction in the anaerobic degradation of the monoterpene mercene to (*S*)-linalool.^189^ In general, however, wild-type variants of hydratases display limited promiscuity, and therefore the synthetic applicability of these enzymes has been severely restricted.^190^

**Scheme 57:**
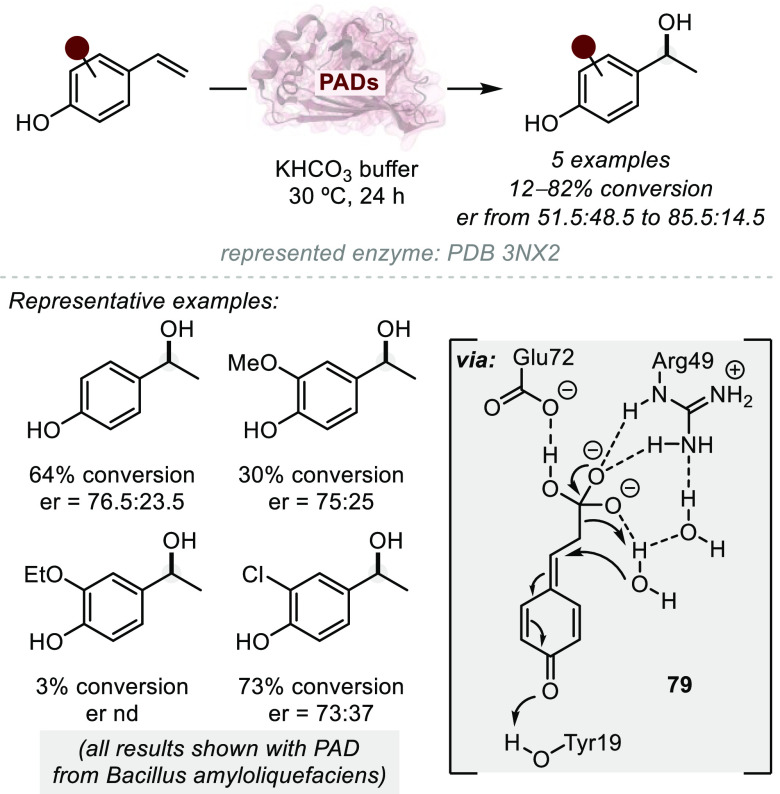
Promiscuous Catalytic Hydration of Hydroxystyrenes with Phenolic Acid Decarboxylates

In 2013, Glueck and Faber et al. serendipitously discovered “hydratase activity” of a variety of phenolic acid decarboxylases and demonstrated formal additions of water to hydroxystyrene derivatives in the presence of carbonate buffer with moderate levels of enantioselectivity (Scheme 57).^191^ The dependency on hydroxystyrenes is explained by the proposed *p*-quinone methide intermediate, an electronically activated motif that undergoes a stereoselective 1,6-addition of water (via TS **79**), a mechanistic feature that severely limits the applicability of the method. Further, the addition of aromatic and alkyl alcohols were later attempted in the presence of ferulic acid decarboxylase from *Enterobacter* sp.; however, none of the tested alcohol nucleophiles underwent addition and only competitive hydration was observed.^192^

**Scheme 58:**
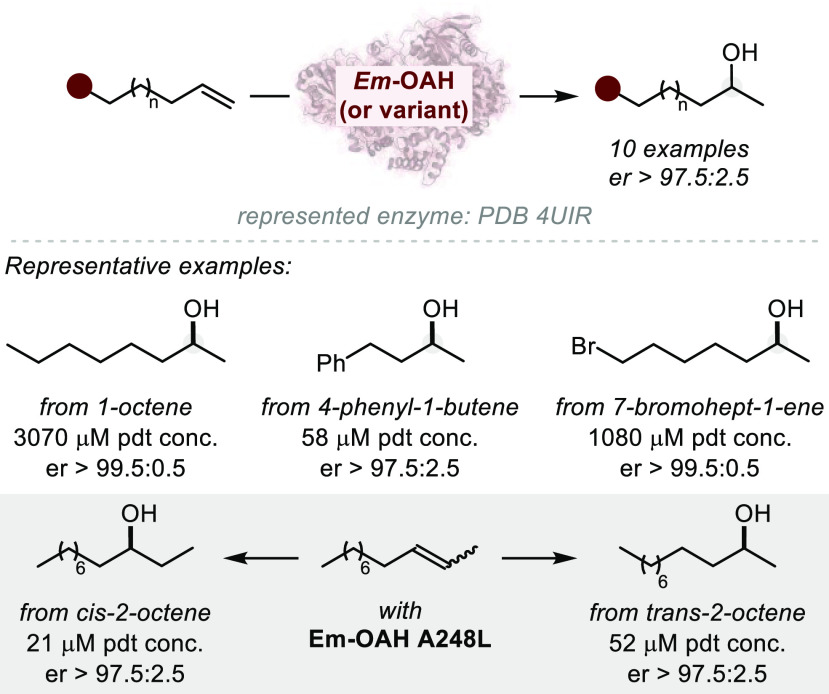
Enzyme-catalyzed Asymmetric Hydration of Simple Alkenes

In 2018, the Hauer group published a groundbreaking study in which they described the promiscuous reactivity of fatty acid hydratases for the hydration of structurally simple C–C double bonds (Scheme 58). In particular, the authors discovered that wild-type oleic acid hydratase (OAH) from *Elizabethkingia meningoseptica*, in the presence of a carboxylic acid decoy molecule catalyzes an asymmetric hydration of 1-decene to form (*S*)-2-decanol in moderate yield (44% isolated, 0.5 mmol) and excellent enantioselectivity (er >99.5:0.5).^193^ Interestingly, the activity of the wild-type enzyme was nearly completely diminished in the presence of shorter chain alkenes, e.g., 1-octene and 1-heptene. To overcome the substrate specificity displayed by the wild type, the authors engineered enzymatic variants capable of the asymmetric hydration of a variety of aliphatic alkenes, including cis- and trans-internal alkenes and the relatively bulky 4-phenyl-1-butene. Impressively, excellent levels of enantioselectivity were observed in all cases. A current drawback of the method is relatively low enzyme activity; however, this discovery nevertheless represents significant progress in the field and shows tremendous promise for future developments in asymmetric additions of oxygen nucleophiles to simple alkenes using engineered enzymes.

## 8 Heterogeneous and Supramolecular Catalysis

Heterogeneous and supramolecular catalysis offer unique possibilities for sustainable chemistry in terms of catalyst recyclability and/or the choice of solvent. In addition to these practical considerations, immobilized or encapsulated active sites can profit from confinement effects that are potentially crucial for product selectivity.^194^ Significant effort has been devoted to the design of heterogeneous conditions that allow catalytic reactivity that is beyond the scope of homogeneous catalysis. Herein we wish to highlight recent endeavors in heterogeneous and supramolecular catalysis within the context of asymmetric hydroalkoxylation and hydroacyloxylation.

**Scheme 59:**
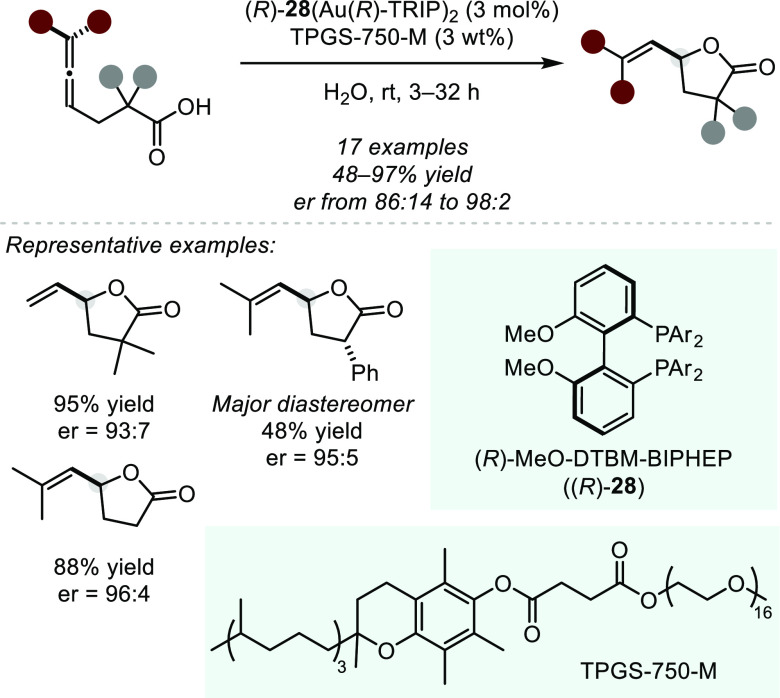
Gold(I)-catalyzed Intramolecular Hydroacyloxylation of Allenes within Nanomicelles

An application of nanomicelles in asymmetric gold catalysis has been published by Lipshutz and co-workers (Scheme 59). The authors utilized the hydrophobic environment within the spontaneously aggregated surfactant TPGS-750-M to enhance tight ion pairing within a cationic (*R*,*R*)–Au(I) complex consisting of a chiral BIPHEP-ligand **28** and an enantiopure TRIP-counteranion to achieve asymmetric intramolecular hydroacyloxylations of allenes.^195^ Notably, the reaction is performed in water at room temperature to give enantioenriched lactones in good yield and enantioselectivity, while an α-substituted racemic allene could be converted to diastereomerically enriched material with high selectivity. Under optimal reaction conditions, additional small amounts of organic solvent (DMSO, benzene, or toluene) were required to achieve short reaction times. As both the catalyst and the surfactant can be recycled, the associated E-factor, a measure of the sustainability of a reaction protocol, is comparably low (4.9). DFT calculations suggest a change in mechanism with regard to the analogous homogeneous gold-catalyzed cyclization of allenic alcohols.^196^ The computed energies for the cyclization mechanism in a model system with PH_3_ as ligand show that the traditional 5-exo-trig cyclization to a protonated lactone is substantially disfavored in comparison to a base-assisted pathway, where initial deprotonation of the carboxylic acid allows almost barrierless product formation. Such change in mechanism gathers further support by the observed rate acceleration in the presence of trimethylamine as additive.

**Scheme 60:**
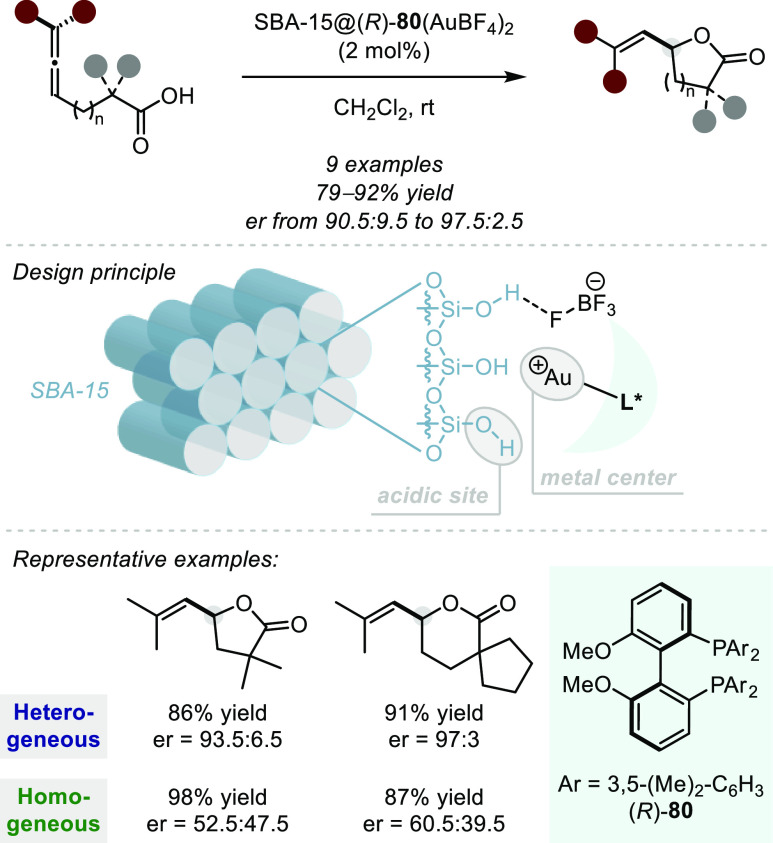
Silica-supported Gold(I)-catalyzed Intramolecular Hydroacyloxylation of Allenes

In addition to their pioneering work in homogeneous gold catalysis, the Toste group has developed heterogeneous conditions for the asymmetric intramolecular hydroacyloxylation of allenes (Scheme 60).^197^ Utilizing a mesoporous silica support material (SBA-15) for cationic gold complexes together with chiral BIPHEP-ligand **78**, the authors were able to obtain lactones in high yield and enantioselectivity. The catalyst design relies on acidic hydroxyl groups within the heterogeneous catalyst to facilitate the crucial protodeauration step that is suggested to be turnover limiting. Therefore, significant rate and selectivity enhancement was observed for the supported catalyst with the regard to corresponding homogeneous conditions. Control experiments with Au catalyst on nonporous support material (pores occupied with cetyltrimethylammonium bromide) suggests that the confined space within the silica pores only plays a minor role in controlling the selectivity. Rather, cooperative hydrogen bonding with the acidic OH groups on the surface is crucial for enantiofacial discrimination. Remarkably, the catalysts could be easily recovered and recycled up to 11 times with no significant loss of selectivity. Stability studies using coupled plasma optical emission spectroscopy (ICP-OES) revealed a total of 3.2% leaching of the gold complex. Further, FTIR spectroscopy shows that a total of 63% molecular catalyst was left after 11 cycles.

**Scheme 61:**
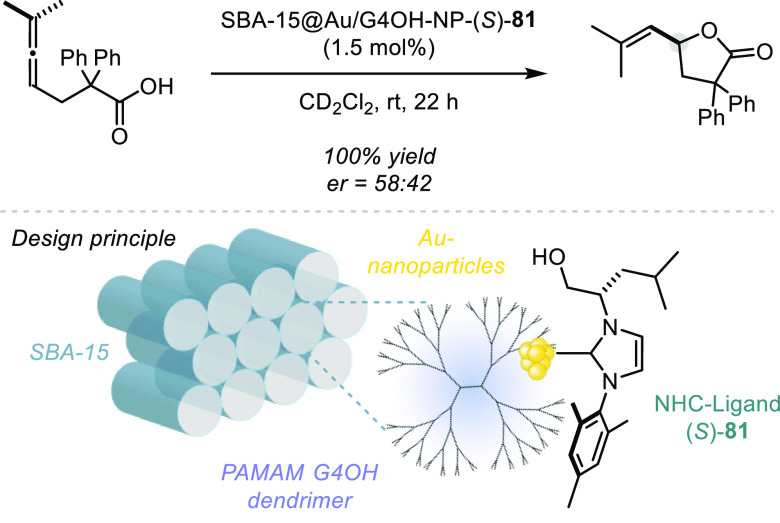
Silica-supported DEMC-catalyzed Intramolecular Hydroacyloxylation of Allenes

A typical observation in gold catalysis is the undesired formation of Au nanoparticles (NP). As for the silica-supported Au catalyst described in Scheme 60, formation of Au-NPs has been observed by the authors after multiple cycles of catalyst recovery. The Toste group therefore attempted to extend the concept of heterogeneous gold catalysis to the purposeful utilization of nanoparticles. Notably, NP-containing catalysts are often unstable toward aggregation or leaching. To circumvent these deleterious effects, the authors designed a catalyst consisting of SBA-15 as mesoporous support material and PAMAM G4OH to form dendrimer-encapsulated metal clusters (DEMCs) together with a chiral NHC ligand **79** (Scheme 61).^198^ The solid catalyst thus obtained consists of highly active aggregation-stable Au-NPs that seemingly possess a surface oxidation state of Au(I) with bound NHC ligands responsible for high activity and induction of stereoselectivity. Despite the poor enantioinduction, this is the first demonstration of stereoselective catalysis by NHC-ligated AU NPs as well as by AU DEMCs. Notably, Au species remaining in solution also show catalytic activity, albeit with no substantial enantioinduction. Additionally, the analogous homogeneous NHC–Au-Cl complex does not catalyze the hydrolactonization while chloride abstraction with AgBF_4_ leads to decomposition and precipitation of Au(0).

A unique approach to combine transition metal and enzyme catalysis has been pioneered by Toste and co-workers.^199^ The supramolecular encapsulation of cationic phosphine–Au(I) complexes in gallium clusters prevents diffusion of the metal into the reaction solution, where it could bind to amino acid residues of the protein such as cysteine, histidine, or asparagine, thus compromising the enzymes activity. The authors where able to demonstrate a tandem enzymatic kinetic resolution and gold-catalyzed intramolecular hydroalkoxylation of allenes with excellent enantioselectivity. However, achiral Me_3_PAu(I) complexes were utilized, consequently resulting in a substrate-controlled hydroalkoxylation with poor diastereomeric selectivity. Yet, the work demonstrates that metal encapsulation can prevent adverse interactions and catalyst deactivation pathways. The extension of the concept to truly asymmetric hydroalkoxylations is anticipated.

## 9 Future Directions and Outlook

The past decade has witnessed a remarkable surge in asymmetric hydroalkoxylation of C–C multiple bonds. The quickening pace of progress in this field using a range of catalytic strategies, aided by its profound synthetic utility, assures the continued vibrancy of this emerging research area in the years to come. Our goal of this section is to identify gaps in the current knowledge and stimulate collective thinking on alternative approaches, which might present avenues for further research.

In particular, asymmetric Markovnikov hydroalkoxylations of unactivated tri- and tetra-substituted alkenes remain out of reach, although reactivity promises to be challenging. Along these lines, considering the wide range of examples shown throughout this review, there is a significant dearth of methods describing asymmetric intermolecular hydroalkoxylations. In fact, only three catalytic methods have displayed this reactivity, each with moderate enantioinduction. Efforts to expand reactivity and stereoselectivity to higher-substituted alkenes, as well as intermolecular systems, is highly warranted. Our group’s work in Brønsted acid-catalyzed intermolecular hydroalkoxylations of styrene has recently opened a new avenues for such reactivity and further developments in this regard can be anticipated.

Despite astounding progress in achieving stereoselectivity for alkenes, asymmetric hydroalkoxylations of cyclopropanes (a well-known alkene surrogate) remain elusive.^200^ Cyclopropanes are prevalent in drug molecules, and their direct functionalization would enable a new strategy for late state functionalization (LSF) as well as study structure–activity relationships (SAR). Looking ahead, we anticipate significant opportunities lie in designing new intra- and intermolecular asymmetric hydroalkoxylation methods of cyclopropanes, cyclopropenes, and vinylcyclopropanes to further expand the repertoire of hydroalkoxylations.

Furthermore, asymmetric anti-Markovnikov additions are still a work in progress. In this regard, harnessing open-shell selectivity presents untold prospects for reaction discovery. Controlling absolute stereochemistry in radical intermediates presents a long-standing challenge, although contemporary efforts have bridled some of the key challenges.^201^ Among different radical-based approaches of designing novel asymmetric anti-Markovnikov hydroalkoxylation, employing photoredox catalysis perhaps holds promise. A significant body of work, spearheaded by Nicewicz and co-workers, leading to the anti-Markovnikov hydrofunctionalization of alkenes have been reported, and initial efforts by Luo and co-workers are highly promising.^140,142^ Similarly, designing new enantioselective PCET-based asymmetric hydrofunctionalization represents another emerging frontier. Given the recent reports of PCET-based hydroalkoxylation^144^ and asymmetric hydroamination^202^ by Knowles and co-workers, we believe the stage is well set for designing new PCET-based asymmetric hydroalkoxylation.

Electrocatalysis represents another enabling tool for taming radical intermediates for asymmetric catalysis. One unique feature of electrochemistry that can be particularly effective in the current context is its ability to dial in precise potential, which allows excellent chemoselectivity among various redox active functionalities. A recent report by Lin and co-workers leveraged electrochemistry to accomplish highly enantioselective hydrocyanation,^203^ highlighting the prospect of these methods to successfully unleash similar hydroalkoxylation reactions. Perhaps a suitable chiral HAT reagent, a chiral photocatalysts, or a chiral mediator could render the above-mentioned open-shell processes asymmetric.

In regard to controlling the stereoselectivity of radical-like intermediates, seminal work by the Pronin^112^ and Shigehisa^113,114^ groups in asymmetric MHAT hydroalkoxylation holds significant promise for the future development of general and highly enantioselective Markovnikov-selective methods. Beyond the practical developments already achieved in this area, the fundamental questions regarding the mechanistic underpinnings of enantioinduction are highly stimulating and offer opportunities for interdisciplinary investigations. We look forward to continued work in this area.

Going beyond the use of a traditional single catalytic activation approach, successful integration of different activation modes has shown to be effective for asymmetric alkene functionalization. Related studies by Melchiorre and co-workers using photo-organocatalytic enantioselective radical cascade reactions (leading to an enantioselective difunctionalization),^204^ and a tandem difunctionalization^205^ by Knowles and co-workers provide a compelling background to design a similar asymmetric hydroalkoxylation using synergistic catalysis.

Another avenue to achieve an anti-Markovnikov hydrofunctionalization is the use of chiral bulky super base. Seminal work by Bandar et al. has shown the successful implementation of super bases in achieving anti-Markovnikov selectivity.^206^ Designing chiral superbase catalysts for the development of asymmetric anti-Markovnikov hydroalkoxylation is therefore ripe for development.

Finally, the synergy among different fields can be judicially harnessed for mechanism-driven reaction development.^207^ As alluded before, the acidity of a given catalyst has remained one of the key considerations in Brønsted acid-catalyzed alkenes activations. The experimental measurement of acidity can inform a suitable catalyst choice for a given transformation.^208^ Acidity of these Brønsted acids can also be computed.^209^ On a similar note, DFT-based methods can also provide valuable impetus in understanding the cooperativity among different catalyst motifs and to facilitate more definitive studies of structure–activity relationship.^210^

While the recent surge in biocatalytic alkene activation has been impressive, its success has suffered due to substrate specificity. With the help of advanced protein engineering techniques such as site-selective mutagenesis, installing new residues has shown to enhance the capabilities of natural enzymes. Theory and machine learning-based methods can play a crucial role in guiding and expediting such processes.^211−213^

Additionally, it seems inevitable that the push to apply site-selective supramolecular catalysis and design asymmetric heterogeneous surface will cause further rapid progress. In this regard, the recent discovery by Tiefenbacher and co-workers using self-assembled resorcin[4]arene hexamer perhaps suits best for an asymmetric development.^214^ We expect that a closer interaction among theory, physical organic study and nanoengineering can play an important role in making these processes more robust.^215−217^ Empowered by mechanistic understanding coupled with increased awareness, more synthetic chemists will likely adopt these unconventional techniques with growing enthusiasm.

By highlighting and conceptualizing many of the recent developments in state-of-the-art asymmetric hydroalkoxylations, we hope that this review will knit together the broad cross-section of computational and synthetic chemists active across various areas of asymmetric catalysis and nucleate new efforts to explore the unknown avenues for further research in this burgeoning area.

## Abbreviations

CPA: chiral phosphoric acid
BIPHEP: bis(phosphanyl)biphenyl
BINOL: 1,1′-bi-2-naphthol
DTBM: 3,5-di-*tert*-butyl-4-methoxyphenyl
IDP: imidodiphosphate
IDPi: imidodiphosphorimidate
LBBA: Lewis base assisted Brønsted acid
LBA: Lewis acid assisted Brønsted acid
NHC: nitrogen heterocyclic carbenes
PMB: *p*-methoxybenzyl
SPINOL: 1,1′-spirobiindane-7,7′-diol
TADDOL: α,α,α′,α′-tetraaryl-1,3-dioxolane-4,5-dimethanol
THF: tetrahydrofuran
THP: tetrahydropyran
